# Dexketoprofen/tramadol 25 mg/75 mg: randomised double-blind trial in moderate-to-severe acute pain after abdominal hysterectomy

**DOI:** 10.1186/s12871-016-0174-5

**Published:** 2016-01-22

**Authors:** R. A. Moore, H. J. McQuay, J. Tomaszewski, G. Raba, D. Tutunaru, N. Lietuviete, J. Galad, L. Hagymasy, D. Melka, J. Kotarski, T. Rechberger, B. Fülesdi, A. Nizzardo, C. Guerrero-Bayón, S. Cuadripani, B. Pizà-Vallespir, M. Bertolotti

**Affiliations:** 1 Pain Research & Nuffield Division of Anaesthetics, University of Oxford, The Churchill, Oxford, UK; 2 Balliol College, University of Oxford, Oxford, UK; 3 Obstetrics-Gynaecology Private Clinic, Bialystok, Poland; 4 Division of Gynaecology, Provincial Hospital in Przemysl, Przemysl, Poland; 5 Genesys Fertility Center, Bucharest, Romania; 6 Gynaecology, Riga East University Hospital Gynaecology Clinic, Riga, Latvia; 7 GYNPOR, s.r.o., Sliac, Slovakia; 8 Gynaecological Department, St. George Fejer County Teaching Hospital, Szekesfehervar, Hungary; 9 Gynaecological Department, Latvian marine Medical Center, Riga, Latvia; 10 I Department of Gynaecological Oncology and Gynaecology, Medical University Hospital No 1, Lublin, Poland; 11 II Department of Gynaecology, Medical University Hospital No 4, Lublin, Poland; 12 Department of Anaesthesiology and Intensive Care, University of Debrecen, Debrecen, Hungary; 13 Clinical Research, Menarini Ricerche S.p.A – Menarini Group, Florence, Italy; 14 Clinical Research, Laboratorios Menarini S.A. – Menarini Group, Badalona, Spain

**Keywords:** Dexketoprofen trometamol, Tramadol, Analgesics, Drug combinations, Pain, Postoperative, Hysterectomy

## Abstract

**Background:**

Dexketoprofen trometamol plus tramadol hydrochloride is a new oral combination of two analgesics, which have different mechanisms of action for the treatment of moderate to severe acute pain.

**Methods:**

Randomised, double-blind, parallel, placebo and active-controlled, single and multiple-dose study to evaluate the analgesic efficacy and safety of dexketoprofen/tramadol 25 mg/75 mg in comparison with the single agents (dexketoprofen 25 mg and tramadol 100 mg) in moderate to severe acute pain after abdominal hysterectomy.

Patients received seven consecutive doses of study drug within a 3-day period, each dose separated by an 8-hour interval. A placebo arm was included during the single-dose phase to validate the pain model.

Efficacy assessments included pain intensity, pain relief, patient global evaluation and use of rescue medication. The primary endpoint was the mean sum of pain intensity differences over the first 8 h (SPID_8_).

**Results:**

The efficacy analysis included 606 patients, with a mean age of 48 years (range 25–73). The study results confirmed the superiority of the combination over the single agents in terms of the primary endpoint (*p* <0.001). Secondary endpoints were generally supportive of the superiority of the combination for both single and multiple doses.

Most common adverse drug reactions (ADRs) were nausea (4.6 %) and vomiting (2.3 %). All other ADRs were experienced by less than 2 % of patients.

**Conclusions:**

The study results provided robust evidence of the superiority of dexketoprofen/tramadol 25 mg/75 mg over the single components in the management of moderate to severe acute pain, as confirmed by the single-dose efficacy, repeated-dose sustained effect and good safety profile observed.

**Trial registration:**

EU Clinical Trials Register (EudraCT number 2012-004545-32, registered 04 October 2012); Clinicaltrials.gov (NCT01904149, registered 17 July 2013).

**Electronic supplementary material:**

The online version of this article (doi:10.1186/s12871-016-0174-5) contains supplementary material, which is available to authorized users.

## Background

In patients with moderate to severe pain, it is difficult to obtain effective analgesia with a single drug and, therefore, analgesic drugs are commonly combined to achieve optimal control of pain [1] as combination of analgesics are often particularly effective [2]. Dexketoprofen trometamol plus tramadol hydrochloride (dexketoprofen/tramadol) is a new oral combination with features of dexketoprofen (fast analgesic effect) and tramadol (long duration of effect) to generate good analgesia at relatively low dosage for the treatment of moderate to severe acute pain.

Dexketoprofen, a non-steroidal anti-inflammatory drug, is a well-known peripheral analgesic drug characterised by a quick onset of effect [3]. The trometamol salt ensures rapid dissolution and absorption with early pain relief [4], which is important for acute pain [5, 6]. Duration of pain relief, however, is limited to 4 or 5 h [7]. Tramadol, μ-opioid receptor agonist, noradrenaline and serotonin re-uptake inhibitor, is a centrally-acting analgesic characterised by a long duration of effect [8].

The combination of dexketoprofen and tramadol is expected to result in balanced peripheral-central analgesia to allow for lower and better tolerated doses than the single agents used alone. In particular, an additive effect of dexketoprofen and tramadol is anticipated to result in adequate analgesia from tramadol at lower doses than recommended on its own for the treatment of moderate to severe pain. In a previous dose-finding trial evaluating four different dose-combinations of dexketoprofen and tramadol versus placebo, the particular combination of dexketoprofen 25 mg plus tramadol 75 mg demonstrated a consistently superior efficacy in all parameters of analgesia tested [9], and it was therefore selected as the optimum combination of doses for further analysis.

The present study aimed to evaluate the analgesic efficacy and safety of the single and repeated-dose administration of dexketoprofen/tramadol 25 mg/75 mg oral fixed combination in comparison with the single agents (tramadol given at a higher dose; 100 mg) in moderate to severe acute pain after abdominal hysterectomy.

It was hypothesised that dexketoprofen/tramadol 25 mg/75 mg would provide a level of analgesia above that achievable by each component alone, without compromising the safety profile.

## Methods

The study (Sponsor Code DEX-TRA-04) was registered at the EU Clinical Trials Register (EudraCT number 2012-004545-32, registered 04 October 2012, URL: https://www.clinicaltrialsregister.eu/ctr-search/search?query=eudract_number%3A2012-004545-32) and at Clinicaltrials.gov, (NCT01904149, registered 17 July 2013, URL: https://www.clinicaltrials.gov/ct2/show/NCT01904149?term=dexketoprofen&rank=3.clinicaltrials.gov). It was performed at 28 study sites in eight European countries (Hungary, Latvia, Lithuania, Poland, Romania, Russian Federation, Slovakia and Spain). It was conducted in accordance with the principles of Good Clinical Practice and the Declaration of Helsinki and was approved by all the concerned Competent Authorities and Ethics Committees (Additional file 1). All participating patients provided written informed consent. The clinical phase of the study started on May 2013 (first patient screened) and concluded on May 2014 (last patient out).

### Patients

Female patients, aged 18 to 75 years, scheduled to undergo a total or subtotal hysterectomy for benign conditions, requiring an infraumbilical laparotomy of ≥3 cm were eligible for the study. Patients were expected to require hospitalisation for at least 3 days after surgery. Criteria for randomisation included postoperative pain of moderate to severe intensity (Visual Analogue Scale [VAS] ≥40) the day after surgery.

Patients were excluded from the study in any of the following circumstances: breastfeeding women, known allergy or contraindication to the study drugs or rescue medication, moderate to severe renal dysfunction, severe hepatic or cardiac dysfunction, history of gastrointestinal disorders, bleeding disorders, asthma, epilepsy, history of drug or alcohol abuse or presence of any medical condition that in the opinion of the investigator might pose a risk to the patient or may confound study results. Concomitant use of analgesics (other than those specified in the protocol) and any medications that could pose a risk to the patient or confound the study results were restricted within a period that depended on the half life of the respective drugs. Further exclusion criteria encompassed surgical complications, chronic opioid use and participation in any other clinical trial within the previous month.

### Study design

It was a randomised, double-blind, double-dummy, parallel, placebo and active-controlled, single and multiple-dose, phase III study, including a total of six treatment arms (Table 1).

**Table 1:** Treatment arms

*Study arm*	*Single-dose phase (first dose)*	*Multiple-dose phase (subsequent six doses)*
1	dexketoprofen/tramadol 25 mg/75 mg	dexketoprofen/tramadol 25 mg/75 mg
2	dexketoprofen 25 mg	dexketoprofen 25 mg
3	tramadol 100 mg	tramadol 100 mg
4	placebo	dexketoprofen/tramadol 25 mg/75 mg
5	placebo	dexketoprofen 25 mg
6	placebo	tramadol 100 mg

The study included a total of six treatment arms, with an imbalanced 3:3:3:1:1:1 allocation ratio

The overall study duration was approximately 6 weeks for each patient, including the *screening period* (within 4 weeks of the randomisation day); the *treatment period* (lasting 3 days) and the *end of study visit* (1 week after the last dose) for final safety follow-up.

The treatment period consisted of a *single-dose phase* (first 8 h after the first dose) followed by a *multiple-dose phase* (subsequent six doses). Each dose of study medication was separated by an 8-hour interval. During the single-dose phase, patients could receive one of four possible treatments (dexketoprofen/tramadol 25 mg/75 mg, dexketoprofen 25 mg, tramadol 100 mg or placebo). During the multiple-dose phase, patients assigned to active treatment were to remain on the same treatment arm while patients assigned to placebo were to be re-allocated to receive one of the three possible active treatments (dexketoprofen/tramadol 25 mg/75 mg, dexketoprofen 25 mg or tramadol 100 mg). Overall, patients were to receive seven consecutive doses of the study drug within a 3-day period.

For those patients who met the selection criteria, the surgical procedure was performed following the study site standard practice. Post-operative analgesia consisted of morphine or other short-acting opioids administered by intravenous or intramuscular route. On the day after surgery, after cessation of the post-operative analgesia, patients experiencing pain of moderate to severe intensity (VAS ≥40) were randomised to receive the assigned study treatment. A limit of 10 a.m. for randomisation was set in order to harmonise the dosing schedule and to allow the last dose on day 1 before midnight.

Participants were randomly assigned to one of six treatment groups (see Table 1) following a blocked randomisation procedure, with a block size of 12 and an imbalanced allocation ratio of 3:3:3:1:1:1. At randomisation, patients were stratified in two categories of initial pain intensity: moderate pain (VAS 40–60) or severe pain (VAS >60). The randomisation process was centralised by an Interactive Voice/Web Response System (IVRS/IWRS) and the treatment code was delivered for each patient according to a computer-generated random allocated sequence (randomisation list) prepared by a Sponsor’s third party prior to the start of the study. Two sets were prepared, one set was used for programming the IVRS/IWRS and the other set was used for the labelling of the study medication. Personnel involved in the preparation or the handling of the randomisation list were not involved in the study conduct and statistical analysis. Participants, healthcare providers, and data collectors involved in the conduct or statistical analysis were unaware of the treatment participants were receiving. Moreover, double-blind conditions were secured by using a double-dummy design; each study dose consisted of one tablet (containing either dexketoprofen/tramadol 25 mg/75 mg, dexketoprofen 25 mg, or placebo) and two capsules (containing either tramadol 50 mg, or placebo).

Rescue medication (metamizole 500 mg, with a maximum recommended daily dose of 2 g) was available on request during the entire treatment period. In addition, during the multiple-dose phase, paracetamol 500 mg could be used as an antipyretic.

### Efficacy evaluation

Following treatment administration, patients were requested to make multiple assessments of pain intensity at rest and on movement (elicited pain upon sitting) and of pain relief on an electronic diary (eDiary) over a period of 3 days, with the last assessment to be recorded 8 h after the last dose of study drug. Patients also had to make an overall assessment of the study medication (patient global evaluation, PGE) at the end of each study phase. The amount and the time when rescue medication was used were also recorded.

Pain intensity was measured on a VAS (0–100) with the left end labelled “no pain” and the right end labelled “worst possible pain” [10, 11], pain relief was measured on a five-point Verbal Rating Scale (VRS) (0 = none, 1 = slight, 2 = moderate, 3 = good, 4 = complete) [10] and the PGE was measured on a five-point VRS (1 = poor, 2 = fair, 3 = good, 4 = very good, 5 = excellent) [10, 12].

At baseline, pain intensity at rest and pain intensity on movement were measured immediately before giving study medication. During the single-dose phase, pain intensity at rest and pain relief were measured at 30 min, 1, 1.5, 2, 3, 4, 6 and 8 h post-dose. During the multiple-dose phase, pain intensity at rest and pain intensity on movement were measured immediately before the administration of each dose of study medication and then every 2 and 4 h, respectively, with the last assessment 8 h after the last dose of study medication, on the third day. Whenever patients used rescue medication or paracetamol, pain intensity and pain relief assessments were recorded (when applicable) immediately before the intake. If a patient withdrew from the study prematurely, the PGE was also requested.

If escape analgesia (rescue medication) was used during the single-dose phase, the *Baseline Observation Carried Forward* method was applied [13], with pain intensity returning to its baseline score and pain relief to zero for all subsequent time points for the next 6 h. If escape analgesia (rescue medication) or paracetamol was used during the multiple-dose phase, the *Last Observation Carried Forward* method was used instead (or the *Worst Observation Carried Forward* method, if the assessment immediately before was missing).

The summed pain intensity differences (SPID) and the total pain relief (TOTPAR) were calculated from the pain intensity (VAS) and pain relief (VRS) scores. SPID was calculated as the time-weighted sum of the pain intensity difference (PID) values from baseline and TOTPAR was calculated as the time-weighted sum of the pain relief scores. The percentages of the theoretical maximum possible SPID (% max SPID) and of the theoretical maximum possible TOTPAR (% max TOTPAR) were also calculated.

The primary efficacy endpoint was the mean SPID at rest over 8 h after the first dose (SPID_8_). The primary efficacy variable was used for the assessment of the co-primary efficacy endpoint to test the superiority of dexketoprofen/tramadol versus dexketoprofen and versus tramadol administered as single agents in the single-dose phase.

Secondary efficacy endpoints during the single and multiple-dose phases included: mean pain intensity (VAS) scores, mean SPID, mean % max SPID, percentage of pain intensity responders (achievement of mean pain intensity (VAS) <40), mean pain relief (VRS) scores, mean TOTPAR, percentage of pain relief responders (achievement of ≥50 % max TOTPAR), use of rescue medication, time to use of rescue medication and PGE at the end of each study phase.

### Safety evaluation

The safety evaluation was based on the incidence, seriousness, intensity and causal relationship of spontaneously reported treatment-emergent adverse events (AEs), i.e. AEs occurred after the first study drug administration. Furthermore, safety was also evaluated by the assessment of clinically significant changes post-dose versus baseline in physical examination, vital signs (blood pressure and heart rate), 12-lead electrocardiogram (ECG) and laboratory safety tests (haematology, biochemistry and urinalysis). Any patient who prematurely withdrew after having received study medication was encouraged to undergo the *end of study visit*.

### Statistical analysis

For the primary efficacy variable, the null hypotheses of equality between dexketoprofen/tramadol and dexketoprofen and between dexketoprofen/tramadol and tramadol in the single-dose phase were tested as co-primary efficacy endpoints using an analysis of covariance (ANCOVA) and a two-sided overall significance level of 5 %. Treatment (as the main effect) and baseline pain intensity (VAS) category were included as covariates. No adjustment for multiplicity was required. Comparison of dexketoprofen and tramadol versus placebo was tested in the same manner to validate the pain model.

Secondary efficacy variables were analysed as follows: pain intensity (VAS), SPID, % max SPID and TOTPAR (quantitative variables) were analyzed analogously to the primary endpoint. Pain relief (VRS) and PGE (ordinal variables) were analyzed by the Wilcoxon rank-sum test. The percentage of responders (for both pain intensity and pain relief) were analysed using a Chi-square test. In addition, the percentage of pain intensity responders over 8 h was analysed using a general estimating equations (GEE) analysis. Time to rescue medication was analyzed using the Kaplan-Meier estimation method and treatment groups were compared using a log-rank test. The percentage of patients using rescue medication was analyzed using a Chi-square test.

It was estimated that a sample size of 600 evaluable patients would be necessary for a power higher than 85 % and a significance level of 0.05 to detect the difference in change of SPID over 8 h from baseline between dexketoprofen/tramadol and each single component. A standard deviation of 94 mm*h and a between difference of at least 35 mm*h was assumed based on previous phase II study data (data on file). It was expected that approximately 800 patients would have to be screened in order to obtain 600 randomised patients, assuming an approximate rate of 25 % screening failures.

## Results

A total of 677 patients were screened, of which 606 patients were randomised and received the first dose of the assigned study treatment, thus constituting the safety population. Efficacy analyses were performed on the “intention-to-treat” (ITT) population of the 606 randomised patients. The “per protocol” (PP) population of 505 patients of the ITT population with no major protocol violations was used to perform confirmatory analyses on the primary endpoint. Patient assignment to the different populations occurred before the study blind was broken (Table 2).

**Table 2:** Analysis populations and patient disposition

N	DKP/TRAM			DKP			TRAM			Overall
	SDP placebo	SDP active	All	SDP placebo	SDP active	All	SDP placebo	SDP active	All	
ITT population	51	152	203	51	151	202	51	150	201	606
Safety population	52	151	203	50	152	202	51	150	201	606
PP population	43	127	170	42	129	171	40	124	164	505

*N* number of patients, *DKP/TRAM* dexketoprofen trometamol/tramadol hydrochloride 25 mg/75 mg, *DKP* dexketoprofen trometamol 25 mg, *TRAM* tramadol hydrochloride 100 mg, *SDP* single-dose phase, *ITT* intention-to-treat, *PP* per protocol. The ITT population included all patients randomised; the safety population included all patients randomised who received at least one dose of study treatment; the PP population included all ITT patients with no major protocol violations

**Fig. 1:**
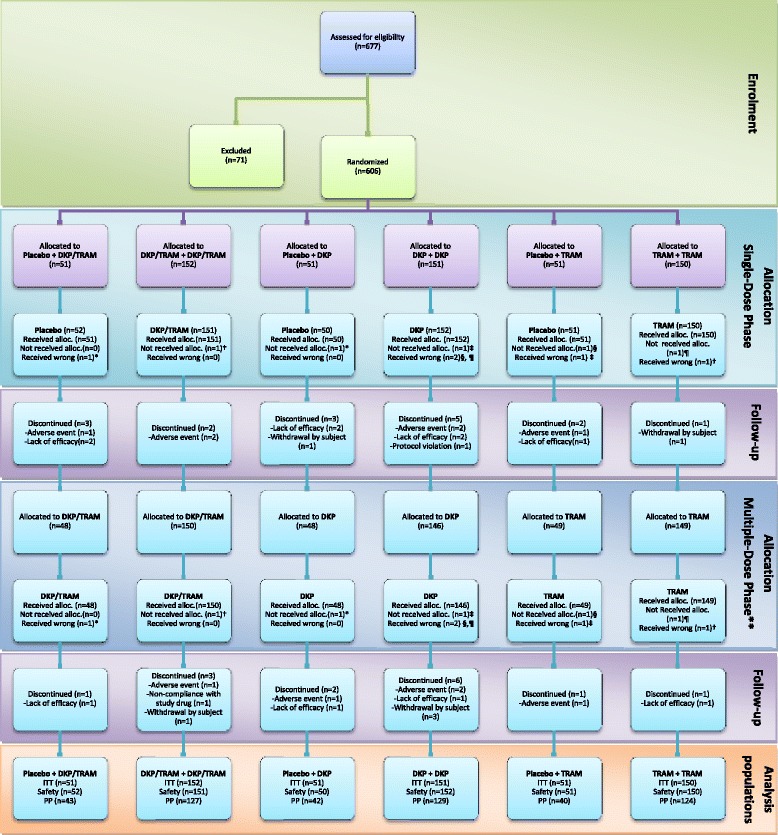
Study CONSORT flow diagram. Participant flow with the numbers of participants who were randomly assigned, received intended treatment, and were analysed for the primary outcome. Five patients in the ITT population received incorrect kit study treatment: *one patient was randomized to receive DKP (first dose placebo) but received DKP/TRAM (first dose placebo) instead; † one patient was randomized to receive DKP/TRAM (first dose active) but received TRAM (first dose active) instead; ‡ one patient was randomized to receive DKP (first dose active) but received TRAM (first dose placebo) instead; § one patient was randomized to receive TRAM (first dose placebo) but received DKP (first dose active) instead; ¶ one patient was randomized to receive TRAM (first dose active) but received DKP (first dose active) instead. ** Received at least one dose; *alloc.* allocated, *ITT* intention-to-treat, *PP* per protocol. The ITT population included all patients randomised; the safety population included all patients randomised who received at least one dose of study treatment; the PP population included all ITT patients with no major protocol violations; *DKP/TRAM* dexketoprofen trometamol/tramadol hydrochloride 25 mg/75 mg, *DKP* dexketoprofen trometamol 25 mg, *TRAM* tramadol hydrochloride 100 mg; n: number of patients

The participant flow with the numbers of participants who were randomly assigned, received intended treatment, and were analysed for the primary outcome is represented in Fig. 1.

Demography and baseline characteristics of different treatment groups were comparable. Demographic and baseline characteristics of the ITT population are represented in Table 3. The overall mean age was 48 years (range 25–73 years). All patients were white. Initial pain was moderate in 38 % patients and severe in 62 % patients.

**Table 3:** Demographic and baseline characteristics (ITT population)

		DKP/TRAM			DKP			TRAM			Overall
		SDP placebo (*N* = 51)	SDP active (*N* = 152)	All (*N* = 203)	SDP placebo (*N* = 51)	SDP active (*N* = 151)	All (*N* = 202)	SDP placebo (*N* = 51)	SDP active (*N* = 150)	All (*N* = 201)	Overall (*N* = 606)
Race N (%)	White	51 (100 %)	152 (100 %)	203 (100 %)	51 (100 %)	151 (100 %)	202 (100 %)	51 (100 %)	150 (100 %)	201 (100 %)	606 (100 %)
Age (years)	mean	47	48	48	47	47	47	47	48	48	48
	SD	6.8	6.7	6.7	7.5	7.1	7.2	4.6	6.9	6.4	6.8
	min	35	25	25	30	31	30	39	32	32	25
	max	69	73	73	71	70	71	61	68	68	73
Height (cm)	mean	165	164	164	164	164	164	164	164	164	164
	SD	6.2	5.4	5.6	5.9	6.4	6.3	5.6	6.1	6.0	5.9
	min	149	152	149	136	151	136	153	149	149	136
	max	175	176	176	175	183	183	176	182	182	183
Weight (kg)	mean	74	75	75	73	72	72	73	71	72	73
	SD	14	15	15	15	14	14	11	12	12	14
	min	52	48	48	50	45	45	50	49	49	45
	max	114	120	120	112	110	112	90	106	106	120
Country	Hungary	8 (16 %)	22 (15 %)	30 (15 %)	7 (14 %)	19 (13 %)	26 (13 %)	7 (14 %)	26 (17 %)	33 (16 %)	89 (15 %)
	Latvia	7 (14 %)	22 (15 %)	29 (14 %)	4 (7.8 %)	17 (11 %)	21 (10 %)	5 (9.8 %)	17 (11 %)	22 (11 %)	72 (12 %)
	Lithuania	1 (2.0 %)	2 (1.3 %)	3 (1.5 %)	1 (2.0 %)	4 (2.6 %)	5 (2.5 %)	3 (5.9 %)	3 (2.0 %)	6 (3.0 %)	14 (2.3 %)
	Poland	18 (35 %)	63 (41 %)	81 (40 %)	23 (45 %)	53 (35 %)	76 (38 %)	17 (33 %)	60 (40 %)	77 (38 %)	234 (39 %)
	Romania	10 (20 %)	32 (21 %)	42 (21 %)	12 (24 %)	40 (27 %)	52 (26 %)	12 (24 %)	32 (21 %)	44 (22 %)	138 (23 %)
	Russian Federation	2 (3.9 %)	3 (2.0 %)	5 (2.5 %)	2 (3.9 %)	3 (2.0 %)	5 (2.5 %)	2 (3.9 %)	3 (2.0 %)	5 (2.5 %)	15 (2.5 %)
	Slovakia	5 (9.8 %)	7 (4.6 %)	12 (5.9 %)	2 (3.9 %)	13 (8.6 %)	15 (7.4 %)	4 (7.8 %)	8 (5.3 %)	12 (6.0 %)	39 (6.4 %)
	Spain	0 (0 %)	1 (0.7 %)	1 (0.5 %)	0 (0 %)	2 (1.3 %)	2 (1.0 %)	1 (2.0 %)	1 (0.7 %)	2 (1.0 %)	5 (0.8 %)
Baseline PI (VAS) N (%)	moderate	19 (37 %)	59 (39 %)	78 (38 %)	19 (37 %)	58 (38 %)	77 (38 %)	19 (38 %)	57 (38 %)	76 (38 %)	231 (38 %)
	severe	32 (63 %)	93 (61 %)	125 (62 %)	32 (63 %)	93 (62 %)	125 (62 %)	31 (62 %)	93 (62 %)	124 (62 %)	374 (62 %)

*ITT* intention-to-treat, *DKP/TRAM* dexketoprofen trometamol/tramadol hydrochloride 25 mg/75 mg, *DKP* dexketoprofen trometamol 25 mg, *TRAM* tramadol hydrochloride 100 mg, *SDP* single-dose phase, *N* number of patients, *SD* standard deviation, *PI* pain intensity, *VAS* visual analogue scale. The ITT population included all randomised patients; PI was measured on a 0–100 VAS with the left end labelled “no pain” and the right end labelled “worst possible pain”

Thirty patients discontinued the study after randomisation (11 patients discontinued due to “lack of efficacy”, 11 patients due to “AEs”, one patient due to “protocol violation”, one patient due to “non-compliance with the study treatment” and six patients due to “withdrawal by patient”), resulting in 576 patients (95 %) completing the study.

### Efficacy results

#### Primary endpoint

**Fig. 2:**
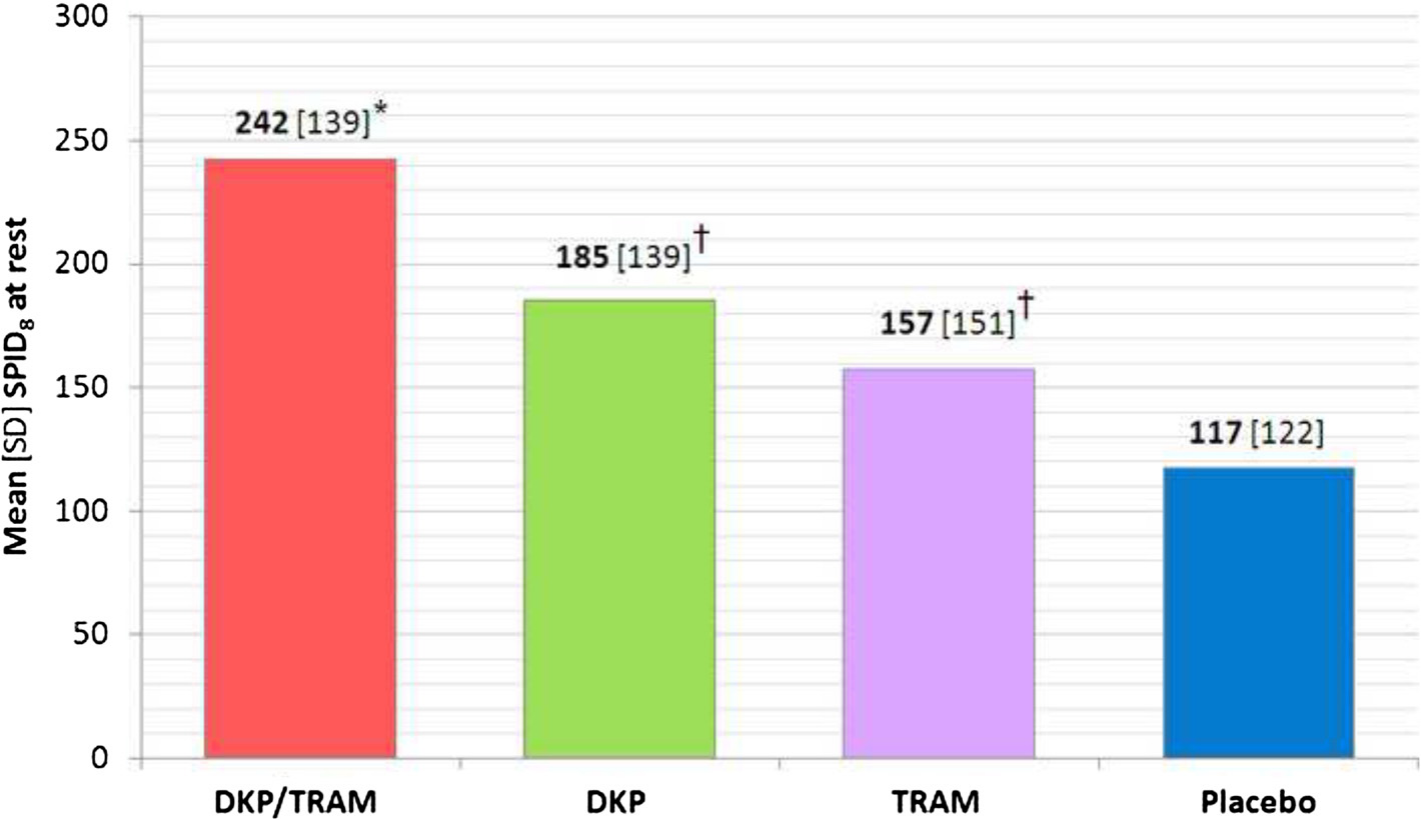
Mean SPID over 8 h (single-dose phase) (Primary Endpoint). *SPID* summed pain intensity differences, *DKP/TRAM* dexketoprofen trometamol/tramadol hydrochloride 25 mg/75 mg, *DKP* dexketoprofen trometamol 25 mg, *TRAM* tramadol hydrochloride 100 mg. Pain intensity (PI) was measured on a 0–100 visual analogue scale (VAS) with left end labelled “no pain” and right end labelled “worst possible pain”; * statistically significant versus both DKP and TRAM (*p* <0.001); † statistically significant versus placebo (*p* <0.05)

The results of the primary analysis (SPID_8_; Fig. 2) confirmed the superiority of dexketoprofen/tramadol over the single components (*p* <0.001). In addition, the comparisons of dexketoprofen and tramadol versus placebo were both statistically significant (*p* <0.001 and *p* = 0.010, respectively), confirming the model sensitivity.

Sensitivity analyses on the PP population confirmed the primary efficacy results. The statistical analysis of SPID_8_ at rest by treatment is presented in Table 4.

**Table 4:** Statistical analysis of SPID_8_ (single-dose phase) (Primary Endpoint) (ANCOVA)

Population		Point Estimate (SE) (Treatment A)	Point Estimate (SE) (Treatment B)	Estimated Treatment Difference (SE) (Treatment A – Treatment B)	95 % CI	*p*-value
Treatment A	Treatment B					
ITT population						
DKP/TRAM	DKP	238 (11)	180 (11)	58 (16)	27 to 88	<0.001
DKP/TRAM	TRAM	238 (11)	153 (11)	85 (16)	54 to 116	<0.001
DKP	Placebo	180 (11)	112 (11)	68 (16)	37 to 99	<0.001
TRAM	Placebo	153 (11)	112 (11)	41 (16)	9.7 to 72	0.010
PP population						
DKP/TRAM	DKP	241 (12)	184 (12)	57 (17)	24 to 90	<0.001
DKP/TRAM	TRAM	241 (12)	160 (12)	81 (17)	48 to 115	<0.001
DKP	Placebo	184 (12)	123 (12)	61 (17)	28 to 95	<0.001
TRAM	Placebo	160 (12)	123 (12)	37 (17)	3.2 to 70	0.032

*SPID*_*8*_ summed pain intensity differences over 8 h post-dose, *ANCOVA* analysis of covariance, *DKP/TRAM* dexketoprofen trometamol/tramadol hydrochloride 25 mg/75 mg, *DKP* dexketoprofen trometamol 25 mg, *TRAM* tramadol hydrochloride 100 mg, *SE* standard error, *CI* confidence interval, *ITT* intention-to-treat, *PP* per protocol. The ITT population included all patients randomised; the PP population included all ITT patients with no major protocol violations; pain intensity (PI) was measured on a 0–100 visual analogue scale (VAS) with the left end labelled “no pain” and the right end labelled “worst possible pain”; SPID was calculated as the time-weighted sum of the pain intensity difference (PID) values from baseline; SPID was tested using an ANCOVA and a two-sided overall significance level of 5 %

#### Secondary endpoints

**Fig. 3:**
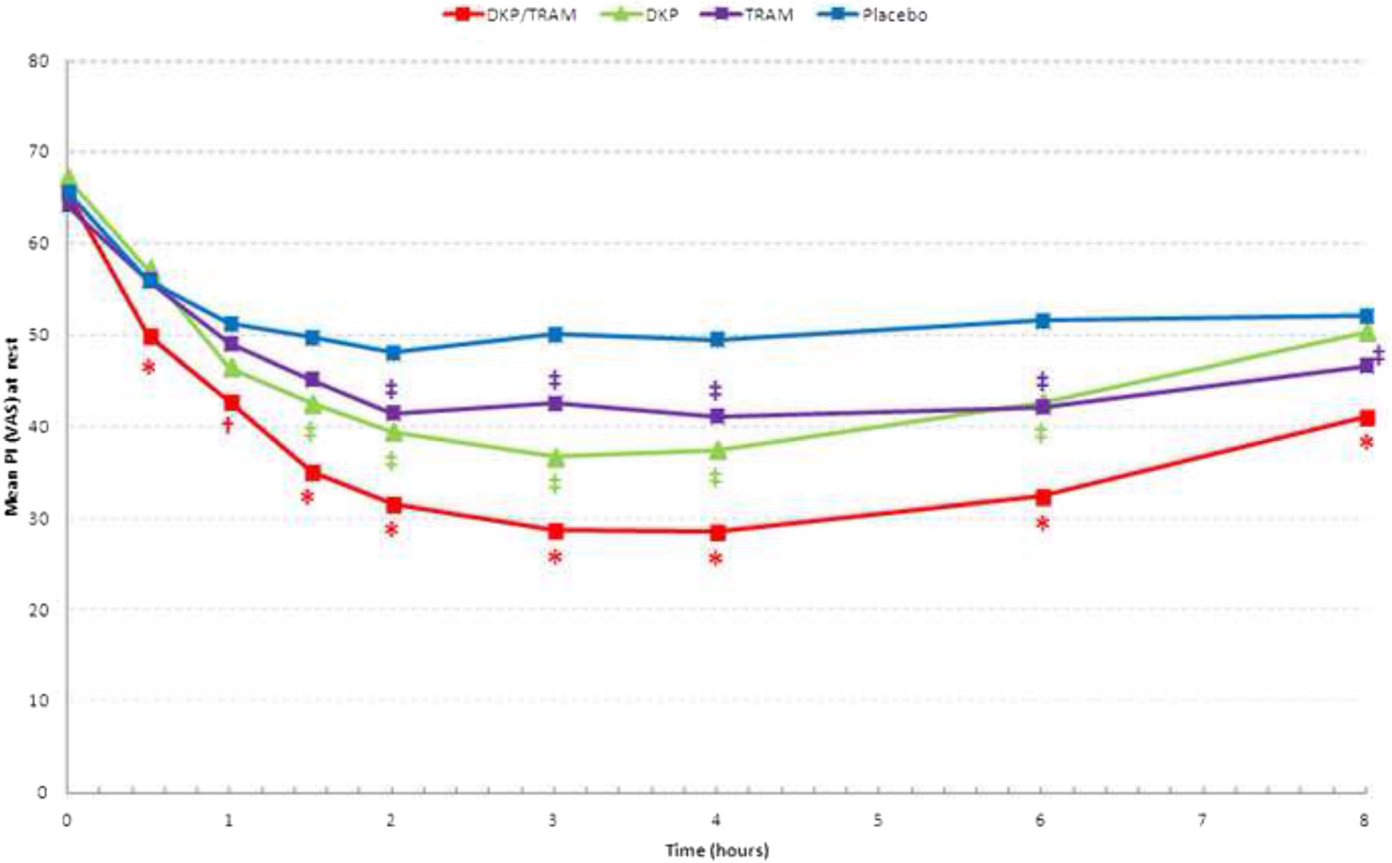
Time course of mean PI (VAS) scores at rest (single-dose phase). *PI* pain intensity, *VAS* visual analogue scale, *DKP/TRAM* dexketoprofen trometamol/tramadol hydrochloride 25 mg/75 mg, *DKP* dexketoprofen trometamol 25 mg, *TRAM* tramadol hydrochloride 100 mg. PI was measured on a 0–100 VAS with left end labelled “no pain” and right end labelled “worst possible pain”; * statistically significant versus both DKP and TRAM (*p* <0.05); † statistically significant versus TRAM only (*p* <0.05); ‡ statistically significant versus placebo (*p* <0.05)

The superiority of dexketoprofen/tramadol over the single components with regards to mean pain intensity (VAS) scores at rest during the single-dose phase was observed at all time points (*p* <0.05), the only exception being dexketoprofen/tramadol over dexketoprofen at the 1-hour time point (*p* = 0.13). The time course of the mean pain intensity (VAS) at rest by treatment during the single-dose phase is represented in Fig. 3.

**Fig. 4:**
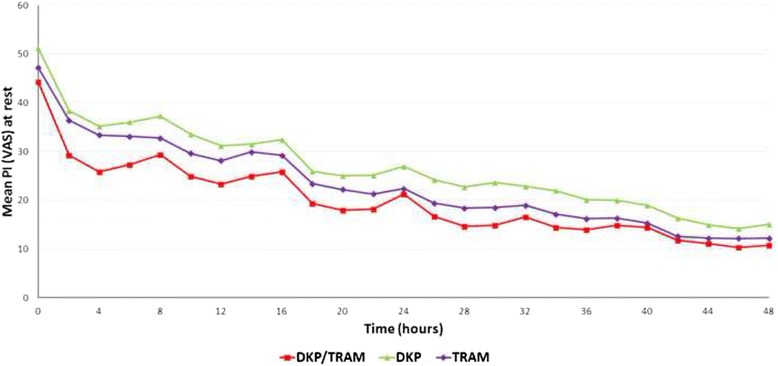
Time course of mean PI (VAS) scores at rest (multiple-dose phase). *PI* pain intensity, *VAS* visual analogue scale, *DKP/TRAM* dexketoprofen trometamol/tramadol hydrochloride 25 mg/75 mg, *DKP* dexketoprofen trometamol 25 mg, *TRAM* tramadol hydrochloride 100 mg. PI was measured on a 0–100 VAS with left end labelled “no pain” and right end labelled “worst possible pain”; statistical significance was achieved versus DKP (*p* = 0.003) over the 48-hour multiple-dose period (*mixed model for repeated measures approach*)

**Fig. 5:**
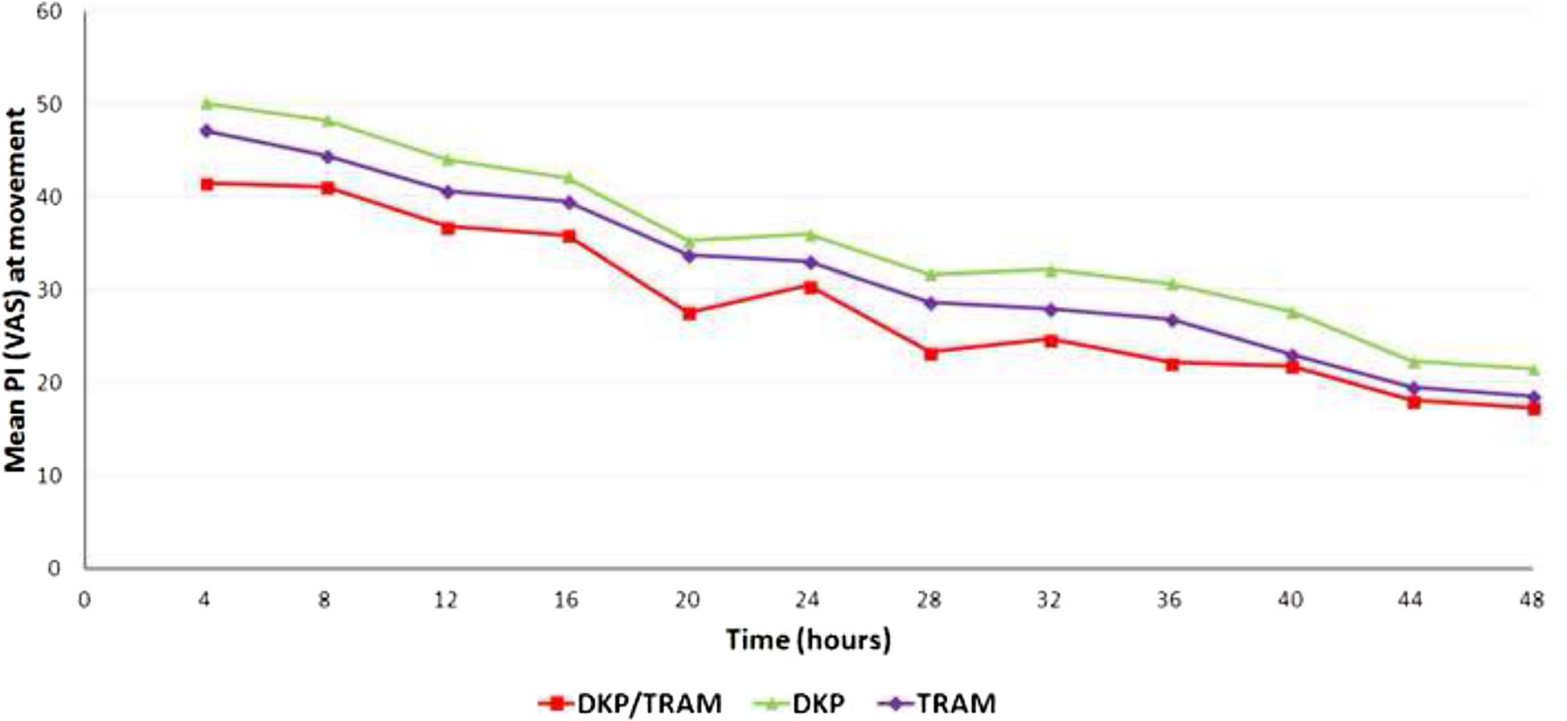
Time course of mean PI (VAS) scores on movement (multiple-dose phase). *PI* pain intensity, *VAS* visual analogue scale, *DKP/TRAM* dexketoprofen trometamol/tramadol hydrochloride 25 mg/75 mg, *DKP* dexketoprofen trometamol 25 mg, *TRAM* tramadol hydrochloride 100 mg. PI was measured on a 0–100 VAS with left end labelled “no pain” and right end labelled “worst possible pain”; pain on movement: elicited pain upon sitting; Statistical significance was achieved versus DKP (*p* <0.001) over the 48-hour multiple-dose period (*mixed model for repeated measures approach)*

During the multiple-dose phase, there was evidence of lower mean pain intensity (VAS) scores at rest over 48 h for dexketoprofen/tramadol than for both single components, with statistical significance achieved over dexketoprofen (*p* = 0.003). Similar results were seen for mean pain intensity (VAS) on movement over 48 h (*p* <0.001 vs. dexketoprofen). A *mixed model for repeated measures* approach was applied for the analysis. The time course of the mean pain intensity (VAS) at rest and on movement by treatment during the multiple-dose phase is represented in Figs. 4 and 5, respectively.

Analyses of mean SPID at rest over 2, 4 and 6 h (SPID_2_, SPID_4_, SPID_6_) and over 24 and 48 h of the multiple-dose phase (SPID_24_ and SPID_48_) confirmed the superiority of dexketoprofen/tramadol over the single components (*p* <0.05). Regarding mean SPID on movement over 24 and 48 h, there was evidence of higher scores on dexketoprofen/tramadol than on dexketoprofen (*p* <0.001).

**Fig. 6:**
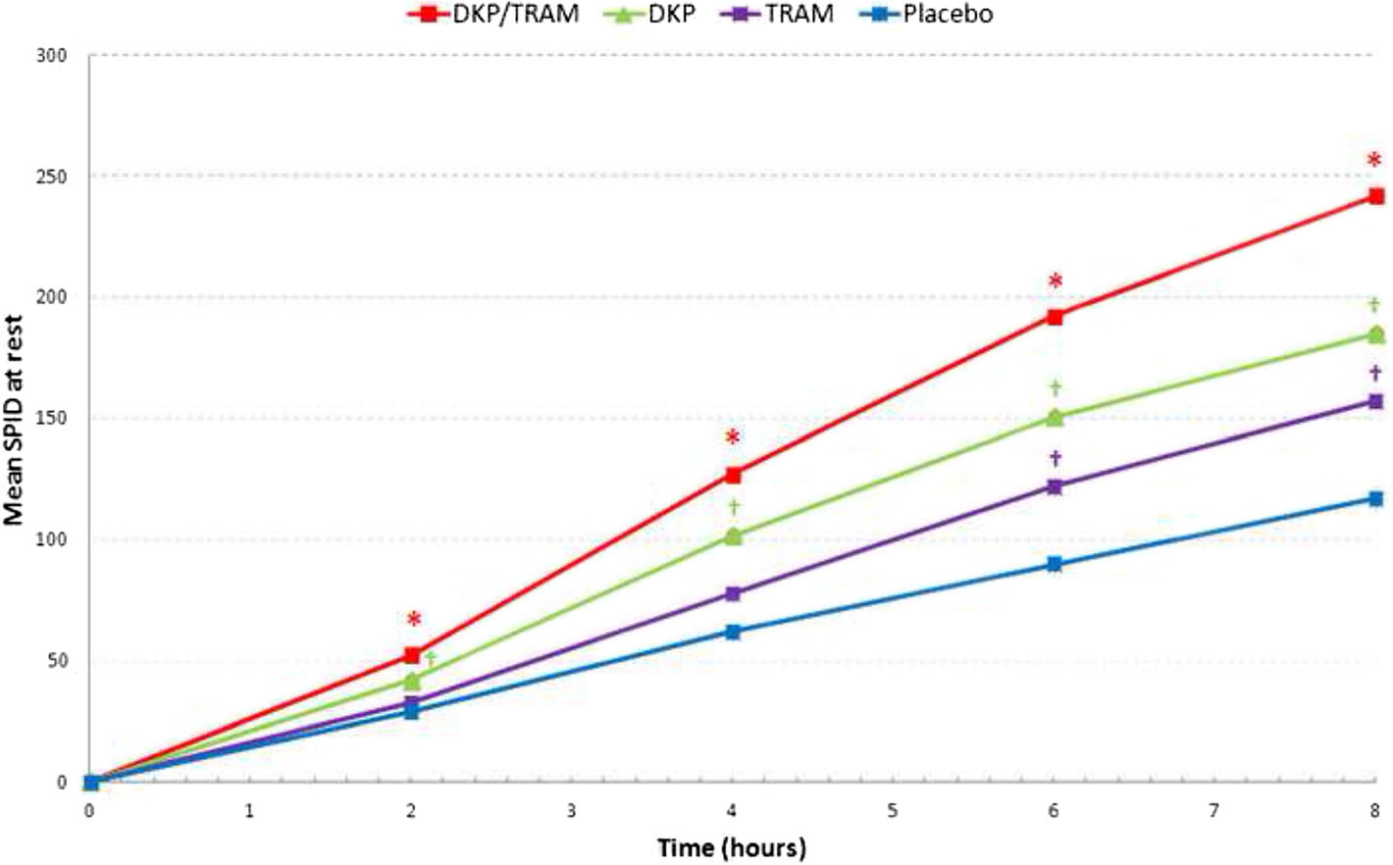
Time course of mean SPID at rest (single-dose phase). *SPID* summed pain intensity differences, *DKP/TRAM* dexketoprofen trometamol/tramadol hydrochloride 25 mg/75 mg, *DKP* dexketoprofen trometamol 25 mg, *TRAM* tramadol hydrochloride 100 mg. Pain intensity (PI) was measured on a 0–100 visual analogue scale (VAS) with left end labelled “no pain” and right end labelled “worst possible pain”; * statistically significant versus both DKP and TRAM (*p* <0.05); † statistically significant versus placebo (*p* <0.05)

Results are presented in Additional file 2 (summary) and Additional file 3 (statistical analysis) for the single-dose phase and in Additional file 4 (summary) and Additional file 5 (statistical analysis) for the multiple-dose phase. The time course of mean SPID at rest during the single-dose phase is represented in Fig. 6.

Similarly, analyses of mean % max SPID at rest over 2, 4, 6, 8 h (% max SPID_2_, % max SPID_4_, % max SPID_6_, % max SPID_8_) and over 24 and 48 h of the multiple-dose phase (% max SPID_24_ and % max SPID_48_), confirmed the superiority of dexketoprofen/tramadol over the single components (*p* <0.05). In addition, analyses of mean % max SPID on movement over 24 and 48 h confirmed the superiority of dexketoprofen/tramadol over the single agents (*p* <0.05).

Results are presented in Additional file 2 (summary) and Additional file 3 (statistical analysis) for the single-dose phase and in Additional file 4 (summary) and Additional file 5 (statistical analysis) for the multiple-dose phase.

There was a significantly higher percentage of pain intensity responders (achievement of mean pain intensity VAS <40 mm) at rest for dexketoprofen/tramadol than for the single components over the first 8 h (65 % for dexketoprofen/tramadol vs. 46 % for dexketoprofen [*p* = 0.001] and 41 % for tramadol [*p* <0.001]). During the multiple-dose phase, the highest percentage of responders at rest over 48 h was achieved with dexketoprofen/tramadol (94 % for dexketoprofen/tramadol vs. 83 % for dexketoprofen [*p* <0.001] and 89 % for tramadol [*p* = 0.048]). Similar results were seen for the response to treatment on movement over 48 h (80 % for dexketoprofen/tramadol vs. 67 % for dexketoprofen [*p* = 0.003] and 71 % for tramadol [*p* = 0.024]). Results are presented in Additional file 6 (single-dose phase) and Additional file 7 (multiple-dose phase).

**Fig. 7:**
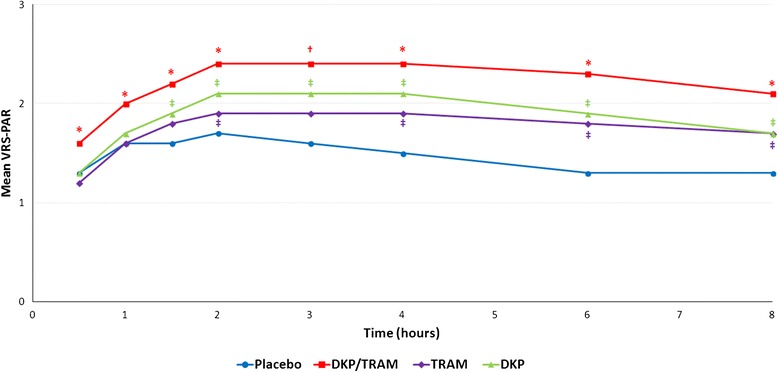
Time course of mean PAR (VRS) scores (single-dose phase). *PAR* pain relief, *VRS* verbal rating scale, *DKP/TRAM* dexketoprofen trometamol/tramadol hydrochloride 25 mg/75 mg, *DKP* dexketoprofen trometamol 25 mg, *TRAM* tramadol hydrochloride 100 mg. PAR was measured on a five-point VRS (0 = none, 1 = slight, 2 = moderate, 3 = good, 4 = complete) during the single-dose phase of the study; * statistically significant versus both DKP and TRAM (*p* <0.05); † statistically significant versus TRAM only (*p* <0.05); ‡ statistically significant versus placebo (*p* <0.05)

The superiority of dexketoprofen/tramadol over the single agents with regards to mean pain relief (VRS) scores during the single-dose phase was observed at all time points (*p* <0.05), with the only exception of dexketoprofen/tramadol over dexketoprofen at the 3-hour time point (*p* = 0.062). The time course of the mean pain relief (VRS) during the single-dose phase is represented in Fig. 7.

The result of the analyses of mean TOTPAR (TOTPAR_2_, TOTPAR_4_, TOTPAR_6_, and TOTPAR_8_) confirmed the superiority of dexketoprofen/tramadol over the single components (*p* <0.05). Results are presented in Additional file 8 (summary) and Additional file 9 (statistical analysis).

The result of the analyses confirmed the superiority of dexketoprofen/tramadol over the single components in terms of the percentage of responders with regards to pain relief (achievement of at least 50 % max TOTPAR; *p* <0.05 for all comparisons). Results are presented in Additional file 10.

There was evidence of a longer overall time to first use of rescue medication on dexketoprofen/tramadol compared with dexketoprofen (*p* = 0.003) and tramadol (*p* = 0.004) as single agents. In addition, dexketoprofen/tramadol was found to be superior to the single components (*p* = 0.008 and *p* = 0.001, respectively) with regards to the time to first use of rescue medication during the single-dose phase.

The percentage of patients using rescue medication over 24 h during the multiple-dose phase was significantly lower with dexketoprofen/tramadol (4.4 %) than with dexketoprofen (11 %; *p* = 0.010) and tramadol (10 %; *p* = 0.021). Results were similar over 48 h (6.4 % versus 12 % and 12 % respectively) and overall (7.9 % versus 13 % and 12 % respectively) during the multiple-dose phase, but the differences did not reach statistical significance.

Dexketoprofen/tramadol was found to be statistically significantly superior to both single components in terms of PGE scores for the single-dose phase (77 % patients in the dexketoprofen/tramadol recorded a “good”, “very good” or “excellent” response compared with 64 % with dexketoprofen [*p* = 0.003] and 67 % patients with tramadol; [*p* <0.001]). Model sensitivity was also confirmed. Results are presented in Additional file 11. However, superiority of dexketoprofen/tramadol over the single agents could not be confirmed for the multiple-dose phase of the study Additional file 12).

### Safety results

Overall, 76 (13 %) patients reported a total of 100 adverse reactions (ADRs), of which 51 were mild, 42 were moderate and seven were severe. The most frequent ADRs (≥2 % amongst the treatment group) were nausea (4.6 % patients; 29 events), vomiting (2.3 % patients; 14 events), abdominal distension (1.5 % patients; nine events), platelet count increased (1.3 % patients; eight events) and blood lactate dehydrogenase increased (1.0 % patients; six events) (Table 5).

**Table 5:** ADRs (active treatment) - by SOC/PT, by treatment group and overall

System organ class	DKP/TRAM *N* = 203	DKP *N* = 202	TRAM *N* = 201	Overall *N* = 606
Preferred term				
Gastrointestinal disorders	13 (6.4 %) | 14	17 (8.4 %) | 18	20 (10 %) | 27	50 (8.3 %) | 59
Nausea	8 (3.9 %) | 9	7 (3.5 %) | 7	13 (6.5 %) | 13	28 (4.6 %) | 29
Vomiting	2 (1.0 %) | 2	6 (3.0 %) | 6	6 (3.0 %) | 6	14 (2.3 %) | 14
Abdominal distension	2 (1.0 %) | 2	1 (0.5 %) | 1	6 (3.0 %) | 6	9 (1.5 %) | 9
Abdominal pain upper	–	2 (1.0 %) | 2	–	2 (0.3 %) | 2
Constipation	1 (0.5 %) | 1	–	1 (0.5 %) | 1	2 (0.3 %) | 2
Dyspepsia	–	2 (1.0 %) | 2	1 (0.5 %) | 1	3 (0.5 %) | 3
Investigations	2 (1.0 %) | 3	11 (5.4 %) | 17	4 (2.0 %) | 4	17 (2.8 %) | 24
Platelet count increased	–	4 (2.0 %) | 4	4 (2.0 %) | 4	8 (1.3 %) | 8
Blood lactate dehydrogenase increased	1 (0.5 %) | 1	5 (2.5 %) | 5	–	6 (1.0 %) | 6
Gamma-glutamyltransferase increased	1 (0.5 %) | 1	3 (1.5 %) | 3	–	4 (0.7 %) | 4
Alanine aminotransferase increased	–	2 (1.0 %) | 2	–	2 (0.3 %) | 2
Blood alkaline phosphatase increased	1 (0.5 %) | 1	1 (0.5 %) | 1	–	2 (0.3 %) | 2
Aspartate aminotransferase increased	–	1 (0.5 %) | 1	–	1 (0.2 %) | 1
Hepatic enzyme increased	–	1 (0.5 %) | 1	–	1 (0.2 %) | 1
Blood and lymphatic system disoders	1 (0.5 %) | 1	1 (0.5 %) | 1	1 (0.5 %) | 1	3 (0.5 %) | 3
Thrombocytosis	1 (0.5 %) | 1	1 (0.5 %) | 1	1 (0.5 %) | 1	3 (0.5 %) | 3
Vascular disorders	2 (1.0 %) | 3	–	–	2 (0.3 %) | 3
Hypertension	1 (0.5 %) | 2	–	–	1 (0.2 %) | 2
Hypertensive crisis	1 (0.5 %) | 1	–	–	1 (0.2 %) | 1
Cardiac disorders	1 (0.5 %) | 1	1 (0.5 %) | 1	–	2 (0.3 %) | 2
Tachycardia	1 (0.5 %) | 1	1 (0.5 %) | 1	–	2 (0.3 %) | 2
Nervous system disorders	–	1 (0.5 %) | 1	1 (0.5 %) | 1	2 (0.3 %) | 2
Dizziness	–	1 (0.5 %) | 1	–	1 (0.2 %) | 1
Headache	–	–	1 (0.5 %) | 1	1 (0.2 %) | 1
Psychiatric disorders	1 (0.5 %) | 1	1 (0.5 %) | 1	–	2 (0.3 %) | 2
Insomnia	–	1 (0.5 %) | 1	–	1 (0.2 %) | 1
Psychotic disorder	1 (0.5 %) | 1	–	–	1 (0.2 %) | 1
Skin and subcutaneous tissue disorders	1 (0.5 %) | 1	–	1 (0.5 %) | 1	2 (0.3 %) | 2
Pruritus	–	–	1 (0.5 %) | 1	1 (0.2 %) | 1
Urticaria	1 (0.5 %) | 1	–	–	1 (0.2 %) | 1
Ear and labyrinth disorders	–	–	1 (0.5 %) | 1	1 (0.2 %) | 1
Vertigo	–	–	1 (0.5 %) | 1	1 (0.2 %) | 1
General disorders and administration site conditions	1 (0.5 %) | 1	–	–	1 (0.2 %) | 1
Asthenia	1 (0.5 %) | 1	–	–	1 (0.2 %) | 1
Metabolism and nutrition disorders	1 (0.5 %) | 1	–	–	1 (0.2 %) | 1
Hypokalaemia	1 (0.5 %) | 1	–	–	1 (0.2 %) | 1
Overall	19 (9.4 %) | 26	30 (15 %) | 39	27 (13 %) | 35	76 (13 %) | 100

*ADR* adverse drug reaction, *SOC* system organ class, *PT* preferred term, *DKP/TRAM* dexketoprofen trometamol/tramadol hydrochloride 25 mg/75 mg, *DKP* dexketoprofen trometamol 25 mg, *TRAM* tramadol hydrochloride 100 mg, *N* number of patients. “Active treatment” refers to events arising after first dose of **active** drug intake (i.e. DKP|TRAM, DKP or TRAM); Results are expressed as number of patients (% of exposed) | number of events

The dexketoprofen/tramadol group presented a lower incidence of ADRs (9.4 % patients) in comparison with the dexketoprofen (15 % patients) and tramadol groups (13 % patients). Overall, 11 (1.8 %) patients reported a total of 15 serious adverse events (SAEs), of which only one (psychotic disorder; in the dexketoprofen/tramadol group) was considered to be treatment-related. There were no marked differences between treatment groups in terms of safety outcomes, including vital signs, physical examination, 12-lead ECG or laboratory safety parameters. It was concluded that the study treatments were safe and well tolerated and that the dexketoprofen/tramadol combination showed a safety profile fully in line with that previously known for the single agents.

## Discussion

The study results confirmed that dexketoprofen/tramadol 25 mg/75 mg is able to provide a level of analgesia above that achievable by each component alone and with an extended duration of effect. Efficacy results were consistent during single and multiple-dose phases, thus supporting the selection of the doses and the regimen proposed, which was based on a previous dose-finding trial [9].

The proposed design was intended to fully characterise the analgesic effect of the fixed combination as a single dose as well as to confirm its sustained efficacy and to evaluate its safety during the repeated administration. Major abdominal surgery is a recognised model of moderate to severe, acute pain, frequently used in the clinical evaluation of analgesic drugs [14–19], though suggested to be of lesser sensitivity than other models such as dental extraction and bunionectomy [20].

A possible limitation of the study was the lack of a sensitivity analysis during the multiple-dose phase. However, the inclusion of a placebo arm during the single-dose phase only was considered as the least burdensome approach for patients.

This was a large trial, conducted across eight different countries and that included multiple investigational sites, where the surgical technique and the anaesthetic regimen could vary. Potential surgical confounders were reduced by limiting the specific procedure type (hysterectomy) and by allowing open procedure only (laparotomy). Furthermore, the restriction to non-malignant conditions additionally ensured the homogeneity of the study population. Other important factors, such as the postoperative analgesic care were standardized, and only those patients experiencing a certain level of pain intensity on the day after surgery were included in the study.

Post-hoc ANCOVA analyses were performed for the primary endpoint including ‘country’ or ‘site’ as additional covariates. A significant (*p* <0.0001) effect, probably related to some low-recruiting sites and countries, was detected in both cases. However, the differences between treatment arms remained significant (*p* <0.01) for all comparisons.

The individual rating of pain intensity is highly subjective and has significant inter- and intra-individual variability. The inclusion of multiple efficacy assessments (pain intensity, pain relief, PGE) and outcomes was aimed to capture the extent of the analgesia that is being produced in the postoperative context. An overall consistency among different outcomes is judged to be more relevant than any particular result.

Pain intensity upon sitting was not recorded during the single-dose phase to avoid unnecessary interferences with pain at rest measurements given that SPID8 at rest was the primary endpoint.

As for the safety results, the combination presented a safety profile fully consistent with what was previously reported for dexketoprofen and tramadol when used as single agents for short term use. No increase of AEs was observed; in fact, the incidence of ADRs with the fixed-dose combination was slightly lower than with both single agents alone.

## Conclusion

The study results provided robust evidence of the efficacy of dexketoprofen/tramadol 25 mg/75 mg in the management of moderate to severe acute pain, as confirmed by the single-dose efficacy, the repeated-dose sustained effect and the good safety profile observed.

## Additional files

Additional file 1: **List of the Ethics Committees that approved the study conduct.** (DOCX 18 kb)

Additional file 2: **Summary of SPID and % max SPID over 2, 4, 6 and 8 h (single-dose phase) (ITT population).** (DOCX 18 kb)

Additional file 3: **Statistical Analysis of SPID and % max SPID over 2, 4, 6 and 8 h (single-dose phase) (ANCOVA) (ITT Population).** (DOCX 18 kb)

Additional file 4: **Summary of SPID and % max SPID at rest and on movement over 24 and 48 h (multiple-dose phase) (ITT Population).** (DOCX 15 kb)

Additional file 5: **Statistical Analysis of SPID and % max SPID at rest and on movement over 24 and 48 h (multiple-dose phase) (ANCOVA) (ITT Population).** (DOCX 15 kb)

Additional file 6: **Percentage of PI (VAS) responders over 8 h (single-dose phase) (ITT Population).** (DOCX 13 kb)

Additional file 7: **Percentage of PI (VAS) responders at rest and on movement over 48 h (multiple-dose phase) (ITT Population).** (DOCX 13 kb)

Additional file 8: **Summary of TOTPAR over 2, 4, 6 and 8 h (single-dose phase) (ITT Population).** (DOCX 14 kb)

Additional file 9: **Statistical Analysis of TOTPAR over 2, 4, 6 and 8 h (ANCOVA) (ITT Population) (single-dose phase).** (DOCX 16 kb)

Additional file 10: **Percentage of responders (≥50 % max TOTPAR) over 2, 4, 6 and 8 h (single-dose phase) (ITT Population).** (DOCX 14 kb)

Additional file 11: **Summary of PGE scores (single-dose phase) (ITT Population).** (DOCX 13 kb)

Additional file 12: **Summary of PGE scores (multiple-dose phase) (ITT Population).** (DOCX 13 kb)
