# Correction to: Dexketoprofen/tramadol 25 mg/75 mg: randomised double-blind trial in moderate-to-severe acute pain after abdominal hysterectomy

**DOI:** 10.1186/s12871-017-0452-x

**Published:** 2017-11-30

**Authors:** R. A. Moore, H. J. McQuay, J. Tomaszewski, G. Raba, D. Tutunaru, N. Lietuviete, J. Galad, L. Hagymasy, D. Melka, J. Kotarski, T. Rechberger, B. Fülesdi, A. Nizzardo, C. Guerrero-Bayón, S. Cuadripani, B. Pizà-Vallespir, M. Bertolotti

**Affiliations:** 1 Pain Research & Nuffield Division of Anaesthetics, University of Oxford, The Churchill, Oxford, UK; 2 Balliol College, University of Oxford, Oxford, UK; 3 Obstetrics-Gynaecology Private Clinic, Bialystok, Poland; 4 Division of Gynaecology, Provincial Hospital in Przemysl, Przemysl, Poland; 5 Genesys Fertility Center, Bucharest, Romania; 6 Gynaecology, Riga East University Hospital Gynaecology Clinic, Riga, Latvia; 7 GYNPOR, s.r.o, Sliac, Slovakia; 8 Gynaecological Department, St. George Fejer County Teaching Hospital, Szekesfehervar, Hungary; 9 Gynaecological Department, Latvian marine Medical Center, Riga, Latvia; 10 Department of Gynaecological Oncology and Gynaecology, Medical University Hospital No 1, Lublin, Poland; 11 II Department of Gynaecology, Medical University Hospital No 4, Lublin, Poland; 12 Department of Anaesthesiology and Intensive Care, University of Debrecen, Debrecen, Hungary; 13 Clinical Research, Menarini Ricerche S.p.A – Menarini Group, Florence, Italy; 14 Clinical Research, Laboratorios Menarini S.A. – Menarini Group, Badalona, Spain

## Correction

Following publication of the original article [1], the authors reported that additional file 10 contained a typing error in the table “Percentage of responders (≥50% max TOTPAR) over two, four, six and eight hours (single-dose phase) (ITT Population)”. The table is to be read as follows:

**Table:** 

		DKP/TRAM(*N* = 152)	DKP (*N* = 151)	TRAM (*N* = 150)	Placebo (*N* = 153)
TOTPAR_2_	Responder, n (%)	91 (60)	69 (46)	58 (39)	50 (33)
	Non-Responder, n (%)	60 (40)	82 (54)	91 (61)	103 (67)
	Treatment comparisons *p*-value				
	DKP/TRAM vs. DKP	0.011			
	DKP/TRAM vs. TRAM	<0.001			
	DKP vs. Placebo	0.020			
	TRAM vs. Placebo	0.258			
TOTPAR_4_	Responder, n (%)	99 (65)	80 (53)	65 (43)	49 (32)
	Non-Responder, n (%)	52 (34)	71 (47)	84 (56)	104 (68)
	Treatment comparisons *p*-value				
	DKP/TRAM vs. DKP	0.026			
	DKP/TRAM vs. TRAM	<0.001			
	DKP vs. Placebo	<0.001			
	TRAM vs. Placebo	0.038			
TOTPAR_6_	Responder, n (%)	105 (69)	72 (48)	64 (43)	49 (32)
	Non-Responder, n (%)	46 (30)	79 (52)	85 (57)	104 (68)
	Treatment comparisons *p*-value				
	DKP/TRAM vs. DKP	<0.001			
	DKP/TRAM vs. TRAM	<0.001			
	DKP vs. Placebo	0.005			
	TRAM vs. Placebo	0.050			
TOTPAR_8_	Responder, n (%)	100 (66)	72 (48)	66 (44)	47 (31)
	Non-Responder, n (%)	51 (34)	79 (52)	83 (55)	106 (69)
	Treatment comparisons *p*-value				
	DKP/TRAM vs. DKP	0.001			
	DKP/TRAM vs. TRAM	<0.001			
	DKP vs. Placebo	0.002			
	TRAM vs. Placebo	0.015			

TOTPAR: total pain relief; % max TOTPAR: percentage of the theoretical maximum possible TOTPAR; ITT: intention-to-treat; DKP/TRAM: dexketoprofen trometamol/tramadol hydrochloride 25 mg/75 mg; DKP: dexketoprofen trometamol 25 mg; TRAM: tramadol hydrochloride 100 mg; N: number of patients; n: number of patients with data. The ITT population included all patients randomised; response was defined as the achievement of at least 50% of the maximum possible TOTPAR within the respective treatment arm; TOTPAR was calculated as the time-weighted sum of the pain relief (PAR) scores; PAR was measured on a five-point verbal rating scale (VRS) (0 = none, 1 = slight, 2 = moderate, 3 = good, 4 = complete); the percentage of PAR responders was analysed using a chi-square test.
