# Supplementary material for: Dexketoprofen/tramadol 25 mg/75 mg: randomised double-blind trial in moderate-to-severe acute pain after abdominal hysterectomy
Source: BMC Anesthesiol. 2016 Jan 22;16:9. doi: 10.1186/s12871-016-0174-5 (PMC4724087; doi:10.1186/s12871-016-0174-5)
Supplement: Supplementary file 1 — List of the Ethics Committees that approved the study conduct. (DOCX 18 kb) [file 12871_2016_174_MOESM1_ESM.docx]

Additional file 1: List of the Ethics Committees that approved the study conduct

| **Country** | Name and Affiliation of the EC | **Type** | **EC originally approved the study protocol:** |
| --- | --- | --- | --- |
| Hungary | Egészségügyi Tudományos Tanács  Klinikai Farmakológiai Etikai  Bizottsága  1051 Budapest, Arany János u. 6-8 | CEC | YES |
| Latvia | Ethics Committee for Clinical Research at Development Society of Pauls Stradins  Clinical University Hospital  Pilsonu street 12, Riga, LV-1002 | CEC | YES |
| Lithuania | Lietuvos bioetikos komitetas  Didzioji g. 22 Vilnius, LT-01128 | CEC | YES |
| Poland | Komisja Bioetyczna przy  Okręgowej Izbie Lekarskiej  W Białymstoku Ul. Świętojańska 7, 15-082, Białystok | CEC | YES |
| Romania | Ministerul Sanatatii  Comisia Natională de Etică Pentru  Studiul Clinical al Medicamentului  011478 București, str. Av Sănătescu nr. 48, sector 1 * | CEC | YES |
| Russia | Ethics committee of GUZ “Moscow Regional Research institute of Obstetrics and Gynecology”  Pokrovka ul., 22 A, Moscow, 101000 | LEC | YES |
|  | Committee on ethics matters at FGBUZ “Clinical hospital #122 n.a. L.G.Sokolov of Federal medico-biological agency”  4, Kultury pr.,Saint Petersburg, Saint Peterburg-194291 | LEC | YES |
| Slovakia | Nezavisla eticka komisia BBSK  Namestie SNP 23, 97401 Banska Bystrica | LEC | YES |
|  | Eticka komisia, Bratislavsky samospravny kraj  Sabinovska 16 82005 Bratislava | LEC | YES |
| Spain | Comité Ético de Investigación Clínica  Hospital de Lleida Arnau de Vilanova  Avda. Alcalde Rovira Roure, 80, Lleida, 25198 | CEC | YES |
|  | Comité Coordinador de Ética de la Investigación Biomédica de Andalucía –  Consejería de Salud Edificio Arena I  Avda. Innovación, s/n Sevilla, 41020 | LEC | N.A. |
|  | Agencia de Ensayos Clínicos Hospital Universitari Vall d'Hebron  Edifici Institut de Recerca, 3ª planta  Passeig Vall d´Hebron, 119-129 Barcelona, 08035 | LEC | N.A. |
|  | Comité Etico de Investigación Clinica de las Islas Baleares (CEIC-IB)  Consellería de Salut I Consum  Camino de Jesús, 38 Palma de Mallorca – Baleares, 07011 | LEC | N.A. |
|  | Comité Ético de Investigación Clínica Subdirección General de Farmacia y Productos Sanitarios  Consellería de Sanidad de Galicia Edificio Administrativo San Lázaro  C/ San Lázaro, s/n Santiago de Compostela - A Coruña, 15703 | LEC | N.A. |
| Ukraine | Komisiia z pytan etyky pry Likarni dlia vchenykh NAN Ukrainy  (Local Ethics Commission of the Hospital for Scientists of the National Academy of Sciences of Ukraine)  vul. Smirnova-Lastochkina, 22, Kyiv, 04053 | LEC | YES |
|  | Komisiia z pytan etyky pry Kyivskomu miskomu tsentri reproduktyvnoi ta perynatalnoi medytsyny*  (Local Ethics Commission of Kyiv City Centre of Reproductive and Perinatal Medicine)  prosp. Heroiv Stalinhrada, 16, Kyiv, 04210 | LEC | YES |
|  | Komisiia z pytan etyky pry Ivano- Frankivskomu oblasnomu perynatalnomu tsentri  (Local Ethics Commission of Ivano-Frankivsk Oblast Perinatal Centre)  vul. Chornovola, 47, Ivano Frankivsk, 76018 | LEC | YES |

CEC: Central Ethics Committe; LEC: Local Ethics; * Changed in 2014 to “Comisia Naţională de Bioetică a Medicamentului şi a Dispozitivelor Medicale, Şos. Ştefan cel Mare nr. 19-21, sector 2, 020125, București”.
