# Supplementary material for: Dexketoprofen/tramadol 25 mg/75 mg: randomised double-blind trial in moderate-to-severe acute pain after abdominal hysterectomy
Source: BMC Anesthesiol. 2016 Jan 22;16:9. doi: 10.1186/s12871-016-0174-5 (PMC4724087; doi:10.1186/s12871-016-0174-5)
Supplement: Supplementary file 2 — Summary of SPID and % max SPID over 2, 4, 6 and 8 h (single-dose phase) (ITT population). (DOCX 18 kb) [file 12871_2016_174_MOESM2_ESM.docx]

Additional file 2: Summary of SPID and % max SPID over two, four, six and eight hours (single-dose phase) (ITT population).

|  | **DKP/TRAM (N=152)** | **DKP  (N=151)** | **TRAM  (N=150)** | **Placebo**  **(N=153)** |
| --- | --- | --- | --- | --- |
| **SPID_2_** | | | | |
| n | 152 | 151 | 150 | 153 |
| Mean (SD) | 52 (37) * | 42 (35) † | 33 (36) | 29 (34) |
| Median (range) | 47 (-22 to 161) | 37 (-30 to 162) | 29 (-55 to 133) | 23 (-52 to 124) |
| **SPID_4_** | | | | |
| n | 152 | 151 | 150 | 153 |
| Mean (SD) | 127 (75) * | 102 (76) † | 77 (76) | 62 (66) |
| Median (range) | 126 (-25 to 333) | 92 (-58 to 320) | 73 (-123 to 267) | 48 (-77 to 270) |
| **SPID_6_** | | | | |
| n | 152 | 151 | 150 | 153 |
| Mean (SD) | 192 (109) * | 150 (111) † | 122 (115) † | 90 (94) |
| Median (range) | 202 (-79 to 460) | 133 (-92 to 476) | 122 (-164 to 394) | 79 (-100 to 368) |
| **SPID_8_ *(Primary Endpoint)*** | | | | |
| n | 152 | 151 | 150 | 153 |
| Mean (SD) | 242 (139) * | 185 (139) † | 157 (151) † | 117 (122) |
| Median (range) | 242 (-79 to 587) | 162 (-100 to 646) | 152 (-251 to 496) | 113 (-118 to 480) |
| **% max SPID_2_** | | | | |
| n | 152 | 151 | 150 | 153 |
| Mean (SD) | 40 (28) * | 32 (27) † | 24 (31) | 22 (27) |
| Median (range) | 37 (-40 to 99) | 29 (-31 to 94) | 21 (-125 to 92) | 16 (-52 to 90) |
| **% max SPID_4_** | | | | |
| n | 152 | 151 | 150 | 153 |
| Mean (SD) | 49 (27) * | 39 (28) † | 29 (33) | 23 (26) |
| Median (range) | 53 (-13 to 96) | 38 (-28 to 93) | 26 (-113 to 93) | 18 (-44 to 90) |
| **% max SPID_6_** | | | | |
| n | 152 | 151 | 150 | 153 |
| Mean (SD) | 49 (27) * | 38 (28) † | 31 (33) † | 23 (25) |
| Median (range) | 51 (-31 to 97) | 37 (-27 to 94) | 29 (-108 to 91) | 19 (-34 to 81) |
| **% max SPID_8_** | | | | |
| n | 152 | 151 | 150 | 153 |
| Mean (SD) | 47 (27) * | 35 (26) † | 29 (35) † | 22 (24) |
| Median (range) | 50 (-35 to 98) | 32 (-26 to 94) | 27 (-194 to 91) | 18 (-32 to 77) |

SPID: summed pain intensity differences; % max SPID: percentage of the theoretical maximum possible SPID; ITT: intention-to-treat; DKP/TRAM: dexketoprofen trometamol/tramadol hydrochloride 25mg/75mg; DKP: dexketoprofen trometamol 25mg; TRAM: tramadol hydrochloride 100mg; N: number of patients; n: number of patients with data; SD: standard deviation. The ITT population included all patients randomised; pain intensity (PI) was measured on a 0-100 visual analogue scale (VAS) with the left end labelled “no pain” and the right end labelled “worst possible pain”; SPID was calculated as the time-weighted sum of the pain intensity difference (PID) values from baseline; * statistically significant versus both DKP and TRAM (p<0.05); † statistically significant versus placebo (p<0.05).
