# Supplementary material for: Dexketoprofen/tramadol 25 mg/75 mg: randomised double-blind trial in moderate-to-severe acute pain after abdominal hysterectomy
Source: BMC Anesthesiol. 2016 Jan 22;16:9. doi: 10.1186/s12871-016-0174-5 (PMC4724087; doi:10.1186/s12871-016-0174-5)
Supplement: Supplementary file 3 — Statistical Analysis of SPID and % max SPID over 2, 4, 6 and 8 h (single-dose phase) (ANCOVA) (ITT Population). (DOCX 18 kb) [file 12871_2016_174_MOESM3_ESM.docx]

Additional file 4: Statistical Analysis of SPID and % max SPID over two, four, six and eight hours (single-dose phase) (ANCOVA) (ITT Population).

| **Time points** | | **Point Estimate (SE)**  **(Treatment A)** | **Point Estimate (SE)**  **(Treatment B)** | **Estimated Treatment Difference (SE) (Treatment A – Treatment B)** | **95% CI** | **p‑value** |
| --- | --- | --- | --- | --- | --- | --- |
| **Treatment A** | **Treatment B** |  |  |  |  |  |
| **SPID_2_** | | | | | | |
| DKP/TRAM | DKP | 51 (2.9) | 41 (2.9) | 11 (4.1) | 2.5 to 19 | 0.011 |
| DKP/TRAM | TRAM | 51 (2.9) | 32 (2.9) | 20 (4.1) | 12 to 28 | <0.001 |
| DKP | Placebo | 41 (2.9) | 28 (2.9) | 13 (4.1) | 4.8 to 21 | 0.002 |
| TRAM | Placebo | 32 (2.9) | 28 (2.9) | 3.8 (4.1) | -4.2 to 12 | 0.352 |
| **SPID_4_** | | | | | | |
| DKP/TRAM | DKP | 125 (5.9) | 100 (6.0) | 25 (8.4) | 9.0 to 42 | 0.002 |
| DKP/TRAM | TRAM | 125 (5.9) | 75 (6.0) | 50 (8.4) | 33 to 66 | <0.001 |
| DKP | Placebo | 100 (6.0) | 60 (6.0) | 40 (8.4) | 24 to 57 | <0.001 |
| TRAM | Placebo | 75 (6.0) | 60 (6.0) | 16 (8.4) | -0.6 to 32 | 0.059 |
| **SPID_6_** | | | | | | |
| DKP/TRAM | DKP | 190 (8.7) | 148 (8.8) | 42 (12) | 18 to 66 | <0.001 |
| DKP/TRAM | TRAM | 190 (8.7) | 119 (8.8) | 71 (12) | 46 to 95 | <0.001 |
| DKP | Placebo | 148 (8.8) | 86 (8.7) | 61 (12) | 37 to 85 | <0.001 |
| TRAM | Placebo | 119 (8.8) | 86 (8.7) | 33 (12) | 8.5 to 57 | 0.008 |
| **SPID_8_** | | | | | | |
| DKP/TRAM | DKP | 238 (11) | 180 (11) | 58 (16) | 27 to 88 | <0.001 |
| DKP/TRAM | TRAM | 238 (11) | 153 (11) | 85 (16) | 54 to 116 | <0.001 |
| DKP | Placebo | 180 (11) | 112 (11) | 68 (16) | 37 to 99 | <0.001 |
| TRAM | Placebo | 153 (11) | 112 (11) | 41 (16) | 9.7 to 72 | 0.010 |
| **% max SPID_2_** | | | | | | |
| DKP/TRAM | DKP | 40 (2.3) | 32 (2.3) | 8.1 (3.3) | 1.7 to 15 | 0.014 |
| DKP/TRAM | TRAM | 40 (2.3) | 25 (2.3) | 16 (3.3) | 9.2 to 22 | <0.001 |
| DKP | Placebo | 32 (2.3) | 22 (2.3) | 9.9 (3.3) | 3.5 to 16 | 0.002 |
| TRAM | Placebo | 25 (2.3) | 22 (2.3) | 2.4 (3.3) | -4.0 to 8.8 | 0.459 |
| **% max SPID_4_** | | | | | | |
| DKP/TRAM | DKP | 49 (2.3) | 39 (2.3) | 9.8 (3.3) | 3.4 to 16 | 0.003 |
| DKP/TRAM | TRAM | 49 (2.3) | 30 (2.4) | 20 (3.3) | 13 to 26 | <0.001 |
| DKP | Placebo | 39 (2.3) | 24 (2.3) | 15 (3.3) | 8.9 to 22 | <0.001 |
| TRAM | Placebo | 30 (2.4) | 24 (2.3) | 5.7 (3.3) | -0.8 to 12 | 0.085 |
| **% max SPID_6_** | | | | | | |
| DKP/TRAM | DKP | 50 (2.3) | 39 (2.3) | 11 (3.3) | 4.5 to 17 | <0.001 |
| DKP/TRAM | TRAM | 50 (2.3) | 31 (2.3) | 19 (3.3) | 12 to 25 | <0.001 |
| DKP | Placebo | 39 (2.3) | 23 (2.3) | 16 (3.3) | 9.2 to 22 | <0.001 |
| TRAM | Placebo | 31 (2.3) | 23 (2.3) | 8.0 (3.3) | 1.6 to 14 | 0.015 |
| **% max SPID_8_** | | | | | | |
| DKP/TRAM | DKP | 50 (2.3) | 36 (2.3) | 11 (3.3) | 4.9 to 18 | <0.001 |
| DKP/TRAM | TRAM | 50 (2.3) | 29 (2.3) | 18 (3.3) | 11 to 24 | <0.001 |
| DKP | Placebo | 36 (2.3) | 23 (2.3) | 13 (3.3) | 6.6 to 19 | <0.001 |
| TRAM | Placebo | 29 (2.3) | 23 (2.3) | 6.8 (3.3) | 0.4 to 13 | 0.037 |

SPID: summed pain intensity differences; % max SPID: percentage of the theoretical maximum possible SPID; ANCOVA: analysis of covariance; ITT: intention-to-treat; SE: standard error; CI: confidence interval; DKP/TRAM: dexketoprofen trometamol/tramadol hydrochloride 25mg/75mg; DKP: dexketoprofen trometamol 25mg; TRAM: tramadol hydrochloride 100mg. The ITT population included all patients randomised; pain intensity (PI) was measured on a 0-100 visual analogue scale (VAS) with the left end labelled “no pain” and the right end labelled “worst possible pain”; SPID was calculated as the time-weighted sum of the pain intensity difference (PID) values from baseline. SPID and % max SPID were tested using an ANCOVA and a two-sided overall significance level of 5%.
