# Supplementary material for: Dexketoprofen/tramadol 25 mg/75 mg: randomised double-blind trial in moderate-to-severe acute pain after abdominal hysterectomy
Source: BMC Anesthesiol. 2016 Jan 22;16:9. doi: 10.1186/s12871-016-0174-5 (PMC4724087; doi:10.1186/s12871-016-0174-5)
Supplement: Supplementary file 4 — Summary of SPID and % max SPID at rest and on movement over 24 and 48 h (multiple-dose phase) (ITT Population). (DOCX 15 kb) [file 12871_2016_174_MOESM4_ESM.docx]

Additional file 3: Summary of SPID and % max SPID at rest and on movement over 24 and 48 hours (multiple-dose phase) (ITT Population).

|  | **DKP/TRAM (N=203)** | **DKP  (N=202)** | **TRAM  (N=201)** |
| --- | --- | --- | --- |
| **SPID_24_ at rest** | | | |
| n | 203 | 202 | 201 |
| Mean (SD) | 1014 (435) * | 839 (479) | 865( 475) |
| Median (range) | 1025 (-456 to 2100) | 866 (-368 to 2030) | 904 (-338 to 2128) |
| **SPID_48_ at rest** | | | |
| n | 203 | 202 | 201 |
| Mean (SD) | 2273 (864) * | 1965 (912) | 2035 (919) |
| Median (range) | 2341 (-912 to 4384) | 2016 (-446 to 4206) | 2126 (-510 to 4374) |
| **SPID_24_ on movement** | |  |  |
| n | 194 | 199 | 196 |
| Mean (SD) | 1018 (499) † | 846 (529) | 919 (517) |
| Median (range) | 1030 (-336 to 2216) | 936 (-816 to 2008) | 956 (-1208 to 2124) |
| **SPID_48_ on movement** | |  |  |
| n | 194 | 199 | 196 |
| Mean (SD) | 2380 (978 ) † | 2051 (1058) | 2212 (997) |
| Median (range) | 2470 (-672 to 4440) | 2252 (-1632 to 4168) | 2260 (-1436 to 4276) |
| **% max SPID_24_ at rest** |  |  |  |
| n | 203 | 202 | 201 |
| Mean (SD) | 64 (24) * | 53 (28) | 55 (29) |
| Median (range) | 68 (-27 to 100) | 54 (-22 to 100) | 58 (-79 to 100) |
| **% max SPID_48_ at rest** |  |  |  |
| n | 203 | 202 | 201 |
| Mean (SD) | 72 (22) * | 62 (25) | 64 (26) |
| Median (range) | 76 (-27 to 100) | 66 (-12 to 100) | 70 (-42 to 100) |
| **% max SPID_24_ on movement** |  |  |  |
| n | 194 | 199 | 196 |
| Mean (SD) | 54 (24) * | 44 (28) | 48 (27) |
| Median (range) | 57 (-23 to 100) | 49 (-52 to 98) | 53 (-103 to 97) |
| **% max SPID_48_ on movement** |  |  |  |
| n | 194 | 199 | 196 |
| Mean (SD) | 63 (22) * | 54 (26) | 58 (24) |
| Median (range) | 68 (-19 to 100) | 60 (-52 to 97) | 64 (-61 to 98) |

SPID: summed pain intensity differences; % max SPID: percentage of the theoretical maximum possible SPID; ITT: intention-to-treat; DKP/TRAM: dexketoprofen trometamol/tramadol hydrochloride 25mg/75mg; DKP: dexketoprofen trometamol 25mg; TRAM: tramadol hydrochloride 100mg; N: number of patients; n: number of patients with data; SD: standard deviation. The ITT population included all patients randomised; pain intensity (PI) was measured on a 0-100 visual analogue scale (VAS) with the left end labelled “no pain” and the right end labelled “worst possible pain”; pain on movement: elicited pain upon sitting; SPID was calculated as the time-weighted sum of the pain intensity difference (PID) values from baseline; * statistically significant versus both DKP and TRAM (p<0.05); † statistically significant versus DKP only (p<0.001).
