# Supplementary material for: Dexketoprofen/tramadol 25 mg/75 mg: randomised double-blind trial in moderate-to-severe acute pain after abdominal hysterectomy
Source: BMC Anesthesiol. 2016 Jan 22;16:9. doi: 10.1186/s12871-016-0174-5 (PMC4724087; doi:10.1186/s12871-016-0174-5)
Supplement: Supplementary file 5 — Statistical Analysis of SPID and % max SPID at rest and on movement over 24 and 48 h (multiple-dose phase) (ANCOVA) (ITT Population). (DOCX 15 kb) [file 12871_2016_174_MOESM5_ESM.docx]

Additional file 5: Statistical Analysis of SPID and % max SPID at rest and on movement over 24 and 48 hours (multiple-dose phase) (ANCOVA) (ITT Population).

| **Time points** | | **Point Estimate (SE)**  **(Treatment A)** | **Point Estimate (SE)**  **(Treatment B)** | **Estimated Treatment Difference (SE) (Treatment A – Treatment B)** | **95% CI** | **p‑value** |
| --- | --- | --- | --- | --- | --- | --- |
| **Treatment A** | **Treatment B** |  |  |  |  |  |
| **SPID_24_ at rest** | | | | | | |
| DKP/TRAM | DKP | 983 (32) | 808 (32) | 175 (44) | 88 to 262 | <0.001 |
| DKP/TRAM | TRAM | 983 (32) | 835 (32) | 148 (44) | 61 to 236 | <0.001 |
| **SPID_48_ at rest** |  |  |  |  |  |  |
| DKP/TRAM | DKP | 2197 (60) | 1887 (60) | 310 (84) | 146 to 474 | <0.001 |
| DKP/TRAM | TRAM | 2197 (60) | 1955 (60) | 242 (84) | 78 to 407 | 0.004 |
| **SPID_24_ on movement** | | | | | | |
| DKP/TRAM | DKP | 998 (37) | 824 (36) | 174 (51) | 74 to 275 | <0.001 |
| DKP/TRAM | TRAM | 998 (37) | 899 (37) | 99 (52) | -2.6 to 200 | 0.056 |
| **SPID_48_ on movement** | | | | | | |
| DKP/TRAM | DKP | 2329 (72) | 1996 (71) | 334 (100) | 138 to 530 | <0.001 |
| DKP/TRAM | TRAM | 2329 (72) | 2159 (72) | 171 (100) | -27 to 368 | 0.090 |
| **% max SPID_24_ at rest** | | | | | | |
| DKP/TRAM | DKP | 64 (1.9) | 53 (1.9) | 11 (2.7) | 5.7 to 16 | <0.001 |
| DKP/TRAM | TRAM | 64 (1.9) | 55 (1.9) | 9.2 (2.7) | 3.8 to 15 | <0.001 |
| **% max SPID_48_ at rest** | | | | | | |
| DKP/TRAM | DKP | 72 (1.7) | 62 (1.7) | 9.5 (2.4) | 4.8 to 14 | <0.001 |
| DKP/TRAM | TRAM | 72 (1.7) | 65 (1.7) | 7.0 (2.4) | 2.3 to 12 | 0.004 |
| **% max SPID_24_ on movement** | | | | | | |
| DKP/TRAM | DKP | 54 (1.9) | 44 (1.9) | 10 (2.7) | 4.7 to 15 | <0.001 |
| DKP/TRAM | TRAM | 54 (1.9) | 48 (1.9) | 6.3 (2.7) | 1.1 to 12 | 0.019 |
| **% max SPID_48_ on movement** | | | | | | |
| DKP/TRAM | DKP | 63 (1.8) | 54 (1.7) | 9.3 (2.4) | 4.5 to 14 | <0.001 |
| DKP/TRAM | TRAM | 63 (1.8) | 58 (1.7) | 5.3 (2.4) | 0.5 to 10 | 0.031 |

SPID: summed pain intensity differences; % max SPID: percentage of the theoretical maximum possible SPID; ANCOVA: analysis of covariance; ITT: intention-to-treat; SE: standard error; CI: confidence interval; DKP/TRAM: dexketoprofen trometamol/tramadol hydrochloride 25mg/75mg; DKP: dexketoprofen trometamol 25mg; TRAM: tramadol hydrochloride 100mg. The ITT population included all patients randomised; pain intensity (PI) was measured on a 0-100 visual analogue scale (VAS) with the left end labelled “no pain” and the right end labelled “worst possible pain”; pain on movement: elicited pain upon sitting; SPID was calculated as the time-weighted sum of the pain intensity difference (PID) values from baseline. SPID and %max SPID were tested using an ANCOVA and a two-sided overall significance level of 5%.
