# Supplementary material for: Dexketoprofen/tramadol 25 mg/75 mg: randomised double-blind trial in moderate-to-severe acute pain after abdominal hysterectomy
Source: BMC Anesthesiol. 2016 Jan 22;16:9. doi: 10.1186/s12871-016-0174-5 (PMC4724087; doi:10.1186/s12871-016-0174-5)
Supplement: Supplementary file 6 — Percentage of PI (VAS) responders over 8 h (single-dose phase) (ITT Population). (DOCX 13 kb) [file 12871_2016_174_MOESM6_ESM.docx]

Additional file 6: Percentage of PI (VAS) responders over eight hours (single-dose phase) (ITT Population).

|  | **DKP/TRAM (N=152)**  **n (%)** | **DKP  (N=151)**  **n (%)** | | **TRAM  (N=150)**  **n (%)** | | **Placebo (N=153)**  **n (%)** |
| --- | --- | --- | --- | --- | --- | --- |
| Responder | 99 (65) | 70 (46) | | 62 (41) | | 48 (31) |
| Non Responder | 53 (35) | 81 (54) | | 88 (59) | | 105 (69) |
|  | | | | | | |
| *Treatment comparisons [*Chi-square test*]* | | | | | *p-value* | |
| DKP/TRAM vs. DKP | | | | | 0.001 | |
| DKP/TRAM vs. TRAM | | | | | <0.001 | |
| DKP vs. Placebo | | | | | 0.007 | |
| TRAM vs. Placebo | | | | | 0.071 | |
| *Treatment comparisons [GEE]* | *Estimate (treatment A / treatment B)* | | *95% CI* | | *p-value* | |
| DKP/TRAM vs. DKP | 2.0 | | 1.4 to 2.8 | | <0.001 | |
| DKP/TRAM vs. TRAM | 2.1 | | 1.4 to 3.1 | | <0.001 | |
| DKP vs. Placebo | 2.0 | | 1.3 to 3.0 | | <0.001 | |
| TRAM vs. Placebo | 1.8 | | 1.2 to 2.8 | | 0.004 | |

PI: pain intensity; VAS: visual analogue scale; ITT: intention-to-treat; DKP/TRAM: dexketoprofen trometamol/tramadol hydrochloride 25mg/75mg; DKP: dexketoprofen trometamol 25mg; TRAM: tramadol hydrochloride 100mg; N: number of patients; n: number of patients with data; CI: confidence interval; GEE: general estimating equation. The ITT population included all patients randomised; PI was measured on a 0-100 VAS with the left end labelled “no pain” and the right end labelled “worst possible pain”; PI response is defined as an achievement of mean PI (VAS) <40; the percentage of PI (VAS) responders were analysed using a Chi-square test; in addition, the percentage of PI responders over 8 hours was analysed using a GEE analysis.
