# Supplementary material for: Dexketoprofen/tramadol 25 mg/75 mg: randomised double-blind trial in moderate-to-severe acute pain after abdominal hysterectomy
Source: BMC Anesthesiol. 2016 Jan 22;16:9. doi: 10.1186/s12871-016-0174-5 (PMC4724087; doi:10.1186/s12871-016-0174-5)
Supplement: Supplementary file 7 — Percentage of PI (VAS) responders at rest and on movement over 48 h (multiple-dose phase) (ITT Population). (DOCX 13 kb) [file 12871_2016_174_MOESM7_ESM.docx]

Additional file 7: Percentage of PI (VAS) responders at rest and on movement over 48 hours (multiple-dose phase) (ITT Population).

|  | **DKP/TRAM (N=203)**  **n (%)** | **DKP  (N=202)**  **n (%)** | **TRAM  (N=201)**  **n (%)** |
| --- | --- | --- | --- |
| **PI (VAS) at rest** |  |  |  |
| Responder | 191 (94) | 167 (83) | 178 (89) |
| Non Responder | 12 (5.9) | 35 (17) | 23 (11) |
| *Treatment comparisons p-value [a]* | | | |
| DKP/TRAM vs. DKP | <0.001 | | |
| DKP/TRAM vs. TRAM | 0.048 | | |
| **PI (VAS) on movement** |  |  |  |
| Responder | 163 (80) | 136 (67) | 142 (71) |
| Non Responder | 40 (20) | 66 (33) | 59 (29) |
| *Treatment comparisons p-value [a]* | | | |
| DKP/TRAM vs. DKP | 0.003 | | |
| DKP/TRAM vs. TRAM | 0.024 | | |

PI: pain intensity; VAS: visual analogue scale; ITT: intention-to-treat; DKP/TRAM: dexketoprofen trometamol/tramadol hydrochloride 25mg/75mg; DKP: dexketoprofen trometamol 25mg; TRAM: tramadol hydrochloride 100mg; N: number of patients; n: number of patients with data. The ITT population included all patients randomised; PI was measured on a 0-100 VAS with the left end labelled “no pain” and the right end labelled “worst possible pain”; pain on movement: elicited pain upon sitting; PI response is defined as an achievement of mean PI (VAS) <40; the percentage of PI (VAS) responders were analysed using a Chi-square test.
