# Supplementary material for: Dexketoprofen/tramadol 25 mg/75 mg: randomised double-blind trial in moderate-to-severe acute pain after abdominal hysterectomy
Source: BMC Anesthesiol. 2016 Jan 22;16:9. doi: 10.1186/s12871-016-0174-5 (PMC4724087; doi:10.1186/s12871-016-0174-5)
Supplement: Supplementary file 8 — Summary of TOTPAR over 2, 4, 6 and 8 h (single-dose phase) (ITT Population). (DOCX 14 kb) [file 12871_2016_174_MOESM8_ESM.docx]

Additional file 8: Summary of TOTPAR over two, four, six and eight hours (single-dose phase) (ITT Population).

|  | **DKP/TRAM**  **(N=152)** | **DKP  (N=151)** | **TRAM  (N=150)** | **Placebo (N=153)** |
| --- | --- | --- | --- | --- |
| **TOTPAR_2_** | | | | |
| n | 151 | 151 | 149 | 153 |
| Mean (SD) | 4.1 (1.7) * | 3.5 (1.8) | 3.3 (1.7) | 3.1 (1.6) |
| Median (range) | 4.0 (0 to 8) | 3.5 (0 to 7) | 3.0 (0 to 8) | 3.0 (0 to 6) |
| **TOTPAR_4_** | | | | |
| n | 151 | 151 | 149 | 153 |
| Mean (SD) | 8.9 (3.2) * | 7.7 (3.6) † | 7.0 (3.6) † | 6.2 (3.3) |
| Median (range) | 10 (0 to 16) | 8.0 (0 to 15) | 7.5 (0 to 16) | 6.5 (0 to 12) |
| **TOTPAR_6_** | | | | |
| n | 151 | 151 | 149 | 153 |
| Mean (SD) | 14 (4.6) * | 11 (5.2) † | 11 (5.5) † | 8.9 (5.1) |
| Median (range) | 15 (0 to 24) | 12 (0 to 23) | 11 (0 to 24) | 9 (0 to 18) |
| **TOTPAR_8_** | | | | |
| n | 151 | 151 | 149 | 153 |
| Mean (SD) | 18 (6.1) * | 15 (6.8) † | 14 (7.5) † | 12 (6.9) |
| Median (range) | 19 (0 to 32) | 16 (0 to 29) | 15 (0 to 32) | 12 (0 to 24) |

TOTPAR: total pain relief; ITT: intention-to-treat; DKP/TRAM: dexketoprofen trometamol/tramadol hydrochloride 25mg/75mg; DKP: dexketoprofen trometamol 25mg; TRAM: tramadol hydrochloride 100mg; N: number of patients; n: number of patients with data; SD: standard deviation. The ITT population included all patients randomised; TOTPAR was calculated as the time-weighted sum of the pain relief (PAR) scores; PAR was measured on a five-point verbal rating scale (VRS) (0=none, 1=slight, 2=moderate, 3=good, 4=complete); * statistically significant versus both DKP and TRAM (p<0.05); † statistically significant versus placebo (p<0.05).
