# Supplementary material for: Dexketoprofen/tramadol 25 mg/75 mg: randomised double-blind trial in moderate-to-severe acute pain after abdominal hysterectomy
Source: BMC Anesthesiol. 2016 Jan 22;16:9. doi: 10.1186/s12871-016-0174-5 (PMC4724087; doi:10.1186/s12871-016-0174-5)
Supplement: Supplementary file 9 — Statistical Analysis of TOTPAR over 2, 4, 6 and 8 h (ANCOVA) (ITT Population) (single-dose phase). (DOCX 16 kb) [file 12871_2016_174_MOESM9_ESM.docx]

Additional file 9: Statistical Analysis of TOTPAR over two, four, six and eight hours (ANCOVA) (ITT Population) (single-dose phase).

| **Time points** | | **Point Estimate (SE)**  **(Treatment A)** | **Point Estimate (SE)**  **(Treatment B)** | **Estimated Treatment Difference (SE) (Treatment A – Treatment B)** | **95% CI** | **p‑value** |
| --- | --- | --- | --- | --- | --- | --- |
| **Treatment A** | **Treatment B** |  |  |  |  |  |
| **TOTPAR_2_** | | | | | | |
| DKP/TRAM | DKP | 4.1 (0.1) | 3.5 (0.1) | 0.6 (0.2) | 0.3-1.0 | 0.001 |
| DKP/TRAM | TRAM | 4.1 (0.1) | 3.3 (0.1) | 0.8 (0.2) | 0.5-1.2 | <0.001 |
| DKP | Placebo | 3.5 (0.1) | 3.2 (0.1) | 0.4 (0.2) | -0.0-0.7 | 0.074 |
| TRAM | Placebo | 3.3 (0.1) | 3.2 (0.1) | 0.1 (0.2) | -0.2-0.5 | 0.446 |
| **TOTPAR_4_** | | | | | | |
| DKP/TRAM | DKP | 8.9 (0.3) | 7.8 (0.3) | 1.1 (0.4) | 0.4-1.9 | 0.004 |
| DKP/TRAM | TRAM | 8.9 (0.3) | 7.1 (0.3) | 1.8 (0.4) | 1.1-2.6 | <0.001 |
| DKP | Placebo | 7.8 (0.3) | 6.3 (0.3) | 1.5 (0.4) | 0.7-2.3 | <0.001 |
| TRAM | Placebo | 7.1 (0.3) | 6.3 (0.3) | 0.8 (0.4) | 0.0-1.6 | 0.045 |
| **TOTPAR_6_** | | | | | | |
| DKP/TRAM | DKP | 14 (0.4) | 12 (0.4) | 2.1 (0.6) | 1.0-3.3 | <0.001 |
| DKP/TRAM | TRAM | 14 (0.4) | 11 (0.4) | 2.9 (0.6) | 1.8-4.1 | <0.001 |
| DKP | Placebo | 12 (0.4) | 9.0 (0.4) | 2.5 (0.6) | 1.4-3.7 | <0.001 |
| TRAM | Placebo | 11 (0.4) | 9.0 (0.4) | 1.7 (0.6) | 0.6-2.9 | 0.004 |
| **TOTPAR_8_** | | | | | | |
| DKP/TRAM | DKP | 18 (0.6) | 15 (0.6) | 3.0 (0.8) | 1.4-4.5 | <0.001 |
| DKP/TRAM | TRAM | 18 (0.6) | 14 (0.6) | 3.7 (0.8) | 2.2-5.3 | <0.001 |
| DKP | Placebo | 15 (0.6) | 12 (0.6) | 3.3 (0.8) | 1.8-4.9 | <0.001 |
| TRAM | Placebo | 14 (0.6) | 12 (0.6) | 2.6 (0.8) | 1.0-4.1 | 0.001 |

TOTPAR: total pain relief; ANCOVA: analysis of covariance; ITT: intention-to-treat; SE: standard error; CI: confidence interval; DKP/TRAM: dexketoprofen trometamol/tramadol hydrochloride 25mg/75mg; DKP: dexketoprofen trometamol 25mg; TRAM: tramadol hydrochloride 100mg. The ITT population included all patients randomised; TOTPAR was calculated as the time-weighted sum of the pain relief (PAR) scores; PAR was measured on a five-point verbal rating scale (VRS) (0=none, 1=slight, 2=moderate, 3=good, 4=complete); TOTPAR was tested using an ANCOVA and a two-sided overall significance level of 5%.
