# Supplementary material for: Dexketoprofen/tramadol 25 mg/75 mg: randomised double-blind trial in moderate-to-severe acute pain after abdominal hysterectomy
Source: BMC Anesthesiol. 2016 Jan 22;16:9. doi: 10.1186/s12871-016-0174-5 (PMC4724087; doi:10.1186/s12871-016-0174-5)
Supplement: Supplementary file 10 — Percentage of responders (≥50 % max TOTPAR) over 2, 4, 6 and 8 h (single-dose phase) (ITT Population). (DOCX 14 kb) [file 12871_2016_174_MOESM10_ESM.docx]

Additional file 10: Percentage of responders (≥50% max TOTPAR) over two, four, six and eight hours (single-dose phase) (ITT Population).

|  |  | **DKP/TRAM (N=152)** | | **DKP  (N=151)** | | **TRAM  (N=150)** | | **Placebo  (N=153)** |
| --- | --- | --- | --- | --- | --- | --- | --- | --- |
| **TOTPAR_2_** | Responder, n (%) | 91 (60) | | 69 (46) | | 58 (39) | | 50 (33) |
|  | Non-Responder, n (%) | 60 (40) | | 82 (54) | | 91 (61) | | 103 (67) |
|  | *Treatment comparisons p-value* | | | | | | | |
|  | DKP/TRAM vs. DKP | 0.011 | | | | | | |
|  | DKP/TRAM vs. TRAM | <0.001 | | | | | | |
|  | DKP vs. Placebo | 0.020 | | | | | | |
|  | TRAM vs. Placebo | 0.258 | | | | | | |
| **TOTPAR_4_** | Responder, n (%) | 99 (65) | 80 (53) | | 65 (43) | | 49 (32) | |
|  | Non-Responder, n (%) | 52 (34) | 71 (47) | | 84 (56) | | 104 (68) | |
|  | *Treatment comparisons p-value* | | | | | | | |
|  | DKP/TRAM vs. DKP | 0.026 | | | | | | |
|  | DKP/TRAM vs. TRAM | <0.001 | | | | | | |
|  | DKP vs. Placebo | <0.001 | | | | | | |
|  | TRAM vs. Placebo | 0.038 | | | | | | |
| **TOTPAR_6_** | Responder, n (%) | 105 (69) | 72 (48) | | 64 (43) | | 49 (32) | |
|  | Non-Responder, n (%) | 46 (30) | 79 (52) | | 85 (57) | | 104 (68) | |
|  | *Treatment comparisons p-value* | | | | | | | |
|  | DKP/TRAM vs. DKP | <0.001 | | | | | | |
|  | DKP/TRAM vs. TRAM | <0.001 | | | | | | |
|  | DKP vs. Placebo | 0.005 | | | | | | |
|  | TRAM vs. Placebo | 0.050 | | | | | | |
| **TOTPAR_8_** | Responder, n (%) | 47 (31) | 100 (66) | | 72 (48) | | 66 (44) | |
|  | Non-Responder, n (%) | 106 (69) | 51 (34) | | 79 (52) | | 83 (55) | |
|  | *Treatment comparisons p-value* | | | | | | | |
|  | DKP/TRAM vs. DKP | 0.001 | | | | | | |
|  | DKP/TRAM vs. TRAM | <0.001 | | | | | | |
|  | DKP vs. Placebo | 0.002 | | | | | | |
|  | TRAM vs. Placebo | 0.015 | | | | | | |

TOTPAR: total pain relief; % max TOTPAR: percentage of the theoretical maximum possible TOTPAR; ITT: intention-to-treat; DKP/TRAM: dexketoprofen trometamol/tramadol hydrochloride 25mg/75mg; DKP: dexketoprofen trometamol 25mg; TRAM: tramadol hydrochloride 100mg; N: number of patients; n: number of patients with data. The ITT population included all patients randomised; response was defined as the achievement of at least 50% of the maximum possible TOTPAR within the respective treatment arm; TOTPAR was calculated as the time-weighted sum of the pain relief (PAR) scores; PAR was measured on a five-point verbal rating scale (VRS) (0=none, 1=slight, 2=moderate, 3=good, 4=complete); the percentage of PAR responders was analysed using a chi-square test.
