# Supplementary material for: Dexketoprofen/tramadol 25 mg/75 mg: randomised double-blind trial in moderate-to-severe acute pain after abdominal hysterectomy
Source: BMC Anesthesiol. 2016 Jan 22;16:9. doi: 10.1186/s12871-016-0174-5 (PMC4724087; doi:10.1186/s12871-016-0174-5)
Supplement: Supplementary file 11 — Summary of PGE scores (single-dose phase) (ITT Population). (DOCX 13 kb) [file 12871_2016_174_MOESM11_ESM.docx]

Additional file 11: Summary of PGE scores (single-dose phase) (ITT Population).

| **Score** | **DKP/TRAM (N=152)  n (%)** | **DKP  (N=151)  n (%)** | **TRAM  (N=150)  n (%)** | **Placebo (N=153) n (%)** |
| --- | --- | --- | --- | --- |
| 1 – Poor | 3 (2.0) | 5 (3.3) | 13 (8.7) | 12 (7.8) |
| 2 – Fair | 21 (14) | 42 (28) | 32 (21) | 58 (38) |
| 3 – Good | 75 (49) | 68 (45) | 78 (52) | 59 (39) |
| 4 – Very Good | 36 (24) | 23 (15) | 18 (12) | 12 (7.8) |
| 5 – Excellent | 6 (3.9) | 5 (3.3) | 4 (2.7) | 2 (1.3) |
|  | | | | |
| *Treatment comparisons p-value* |  |  |  |  |
| DKP/TRAM vs. DKP | 0.003 |  |  |  |
| DKP/TRAM vs. TRAM | <0.001 |  |  |  |
| DKP vs. placebo | 0.001 |  |  |  |
| TRAM vs. placebo | 0.007 |  |  |  |

PGE: patient global evaluation; ITT: intention-to-treat; DKP/TRAM: dexketoprofen trometamol/tramadol hydrochloride 25mg/75mg; DKP: dexketoprofen trometamol 25mg; TRAM: tramadol hydrochloride 100mg; N: number of patients; n: number of patients with data. The ITT population included all patients randomized; PGE was measured on a five-point verbal rating scale (VRS) (1=poor, 2=fair, 3=good, 4=very good, 5=excellent); PGE was analysed by the Wicoxon rank-sum test.
