# Supplementary material for: Dexketoprofen/tramadol 25 mg/75 mg: randomised double-blind trial in moderate-to-severe acute pain after abdominal hysterectomy
Source: BMC Anesthesiol. 2016 Jan 22;16:9. doi: 10.1186/s12871-016-0174-5 (PMC4724087; doi:10.1186/s12871-016-0174-5)
Supplement: Supplementary file 12 — Summary of PGE scores (multiple-dose phase) (ITT Population). (DOCX 13 kb) [file 12871_2016_174_MOESM12_ESM.docx]

Additional file 12: Summary of PGE scores (multiple-dose phase) (ITT Population).

| **Time point**  **Score** | **DKP/TRAM (N=203)  n (%)** | **DKP  (N=202)  n (%)** | **TRAM  (N=201)  n (%)** |
| --- | --- | --- | --- |
| **Day 3** | | | |
| 1 – Poor | 0 | 1 (0.5) | 1 (0.5) |
| 2 – Fair | 9 (4.4) | 9 (4.5) | 11 (5.5) |
| 3 – Good | 39 (19) | 53 (26) | 64 (32) |
| 4 – Very Good | 97 (48) | 75 (37) | 75 (37) |
| 5 – Excellent | 35 (17) | 33 (16) | 33 (16) |
|  | | | |
| *Treatment comparisons p-value* |  |  |  |
| DKP+TRAM vs. DKP | 0.160 |  |  |
| DKP+TRAM vs. TRAM | 0.026 |  |  |

PGE: patient global evaluation; ITT: intention-to-treat; DKP/TRAM: dexketoprofen trometamol/tramadol hydrochloride 25mg/75mg; DKP: dexketoprofen trometamol 25mg; TRAM: tramadol hydrochloride 100mg; N: number of patients; n: number of patients with data. The ITT population included all patients randomized; PGE was measured on a five-point verbal rating scale (VRS) (1=poor, 2=fair, 3=good, 4=very good, 5=excellent); PGE was analysed by the Wicoxon rank-sum test.
